# Effectiveness of mHealth Interventions Targeting Health Care Workers to Improve Pregnancy Outcomes in Low- and Middle-Income Countries: A Systematic Review

**DOI:** 10.2196/jmir.5533

**Published:** 2016-08-19

**Authors:** Mary Amoakoh-Coleman, Alexander Berend-Jan Borgstein, Stephanie FV Sondaal, Diederick E Grobbee, Andrea Solnes Miltenburg, Mirjam Verwijs, Evelyn K Ansah, Joyce L Browne, Kerstin Klipstein-Grobusch

**Affiliations:** ^1^ Julius Global Health, Julius Center for Health Sciences and Primary Care University Medical Center Utrecht University, Utrecht, Netherlands Utrecht Netherlands; ^2^ School of Public Health Epidemiology and Disease Control University of Ghana Accra Ghana; ^3^ Institute of Health and Society, Department of Community Medicine, University of Oslo, Norway Oslo Norway; ^4^ International Institute for Communication and Development The Hague Netherlands; ^5^ Afya (4) Connect Change Lake Zone Mwanza United Republic Of Tanzania; ^6^ Ghana Health Sevice Research and Development Division Accra Ghana; ^7^ Julius Global Health, Julius Center for Health Sciences and Primary Care University Medical Center, Utrecht University, Utrecht, Netherlands Utrecht Netherlands; ^8^ School of Public Health, Faculty of Health Sciences Division of Epidemiology and Biostatistics University of the Witwatersrand Johannesburg South Africa

**Keywords:** maternal, mHealth, neonatal, providers of care, low- and middle-income countries

## Abstract

**Background:**

Low- and middle-income countries (LMICs) face the highest burden of maternal and neonatal deaths. Concurrently, they have the lowest number of physicians. Innovative methods such as the exchange of health-related information using mobile devices (mHealth) may support health care workers in the provision of antenatal, delivery, and postnatal care to improve maternal and neonatal outcomes in LMICs.

**Objective:**

We conducted a systematic review evaluating the effectiveness of mHealth interventions targeting health care workers to improve maternal and neonatal outcomes in LMIC.

**Methods:**

The Cochrane Library, PubMed, EMBASE, Global Health Library, and Popline were searched using predetermined search and indexing terms. Quality assessment was performed using an adapted Cochrane Risk of Bias Tool. A strength, weakness, opportunity, and threat analysis was performed for each included paper.

**Results:**

A total of 19 studies were included for this systematic review, 10 intervention and 9 descriptive studies. mHealth interventions were used as communication, data collection, or educational tool by health care providers primarily at the community level in the provision of antenatal, delivery, and postnatal care. Interventions were used to track pregnant women to improve antenatal and delivery care, as well as facilitate referrals. None of the studies directly assessed the effect of mHealth on maternal and neonatal mortality. Challenges of mHealth interventions to assist health care workers consisted mainly of technical problems, such as mobile network coverage, internet access, electricity access, and maintenance of mobile phones.

**Conclusions:**

mHealth interventions targeting health care workers have the potential to improve maternal and neonatal health services in LMICs. However, there is a gap in the knowledge whether mHealth interventions directly affect maternal and neonatal outcomes and future research should employ experimental designs with relevant outcome measures to address this gap.

## Introduction

The risk for maternal or newborn death is considerably higher in low- and middle-income countries (LMICs) as compared with high-income countries. Despite progress with global decline in maternal mortality, many LMICs still have high maternal mortality rates [1] in particular in LMICs in sub-Saharan Africa and Asia, where the majority of maternal deaths occur [2]. Between 1990 and 2010, globally the under-five mortality rate was reduced by only 28% instead of the targeted 67% [3,4]. Neonatal mortality rate counts toward 41% of the total under-five mortality rate and plays an important role in the slow reduction of under-five mortality rate.

High neonatal mortality rate particularly persists in LMICs [4]. This high burden of maternal and neonatal deaths is compounded with low numbers of physicians and midwives [4]. These human resource challenges are worsened by factors including migration of qualified health workers to better resourced countries, inadequate investment in national health systems, and devastation by major epidemics such as human immunodeficiency virus (HIV) and acquired immune deficiency syndrome (AIDS), and malaria [5]. Task shifting as well as innovation in service delivery using available technology provides a promising opportunity to improve maternal and neonatal outcomes [6].

A potential tool to address maternal and neonatal outcome in LMICs is provided by the global increase in mobile technology. The International Telecommunication Union reported that in 2013, global mobile-phone subscriptions reached 6.8 billion and that the mobile-cellular penetration rate or the number of active mobile phone users within a specific population reached 89% in developing countries [7]. This has facilitated the development of a new component of electronic health, namely mobile health (mHealth). The main feature of mHealth is the exchange of health-related information in the form of coded data, text, images, audio, and video using mobile devices. This technology can be used to address challenges such as access, quality, affordability, matching of resources, and behavioral norms [8]. mHealth can be used by health care workers in LMICs to improve affordability of interventions for health promotion, increase health education, and address disease prevention [9-12]. mHealth could also play a prominent role in task shifting, allowing health care workers closely related to the community, such as community health workers (CHWs), to become an important intermediate between higher health institutions and the community. Although the global use of mHealth interventions is increasing, evidence of the effectiveness of mHealth apps is mostly limited to high-income countries, with a focus on the prevention and management of classic chronic diseases, such as diabetes and hypertension [13]. In LMICs, mHealth interventions have been successfully implemented for the management of HIV and tuberculosis [14-16]. Less evidence exists on the effectiveness of mHealth interventions aimed at health care workers providing maternal and neonatal services in LMICs. Therefore, the main objective of this study was to perform a systematic review to assess the effectiveness of mHealth interventions aimed at health care workers providing maternal and neonatal services in improving maternal and neonatal outcomes in LMIC.

## Methods

### Protocol and Registration

The current systematic review is based on the guidelines provided by PRISMA [17] and was registered in the PROSPERO registry for systematic reviews: (CRD42014010292). This review is part of a larger systematic review that investigated the potential of mHealth interventions targeting both health workers and pregnant women in LMICs to improve maternal and neonatal outcomes.

### Information Sources and Search

An electronic systematic literature search was conducted within the following 5 databases: The Cochrane Library (Cochrane Database of Systemic Reviews), PubMed or MEDLINE, EMBASE, Global Health Library, and POPLINE using predefined search terms (Title or Abstract) and indexing terms (MeSH, Emtree) during the period of June 1, 2014, and August 31, 2014. In addition, Grey literature search was performed between October 2014 and April 2015 because many studies focusing on mHealth interventions are not published in peer-reviewed journals. A list was created of organizations working with mHealth interventions. These organizations consisted of nongovernmental organizations, governments’ agencies, and the World Health Organization working group on mHealth (Multimedia Appendix 1). The websites of these organizations were searched for publications fitting the eligibility criteria. Furthermore, personal contacts (met through working in the field or at conferences) were approached for papers or documents to be included. Additional papers were found via the snowballing effect, using the reference list of included papers.

### Eligibility Criteria

Studies focusing on the domain health care workers in combination with maternal and neonatal care in LMICs were eligible for inclusion. The list of LMICs was created according to the World Bank Classification [12]. The determinant mHealth was defined as a medical and public health practice supported by mobile devices, such as mobile phones, tablets, and other wireless devices [12,18,19]. It makes use of voice messaging, short messaging service (SMS) text messaging, and apps that can be accessed via general packet radio service, third and fourth generation mobile telecommunications (3G and 4G systems), global positioning system, and Bluetooth technology. The outcomes were not prespecified because of the interest for any outcome related to our domain and determinant. Keywords used in these searches included pregnancy, pregnant, midwife, midwives, traditional midwives, traditional birth attendants (TBAs), CHW, maternal, antenatal, delivery, postnatal, neonatal, perinatal, baby, low resource setting, constrained resource, mHealth, mobile phone, smartphone, mobile app, tablet computer, SMS, short messaging, and telemedicine. The full search strategy can be found in Multimedia Appendix 2.

Included papers were all peer-reviewed, written in English, Dutch, French, German, or Spanish, and primary study papers. Papers were excluded when they did not match the domains and determinants, or were reports of proceedings, project protocols, secondary analysis, animal, biomolecular, or genetic studies. Citations of secondary analysis were reviewed for relevant citations. Interventions relating to the termination of pregnancy were excluded when they targeted the termination of pregnancy before 26-week gestation, as the fetus is then not yet regarded as viable. Interventions making use of a radio were excluded because these interventions fell outside the scope of our definition of mHealth.

### Study Selection

The database searches were carried out by ABB and SFS. Subsequent review of search results was undertaken by ABB, MAC, SFS, JB, and KKG. Three reviewers (ABB, ASM, and MV) screened the papers found in the grey literature search. There were no disagreements on paper inclusion.

### Data Extraction

Data extraction was done according to a standardized data extraction form based on: the study, study design, location, target population or size, form of mHealth, focus of evaluation measure (whether maternal or neonatal), mHealth function, relevant study findings with respect to outcome used in the study, role of mHealth, and the strengths, weaknesses, opportunities, and threats of the intervention.

Extraction of the data from database papers was done by a single reviewer (ABB) who was not blinded for journal or author details. Lack of clarity during the extraction process was resolved by consulting the second reviewer (MAC). Data extraction of the grey literature was done by 4 reviewers (ABB, ASM, MV, and MAC). In case of incomplete data, one attempt was made to contact the corresponding author by email.

### Quality Assessment

The quality of the included papers was assessed according to an adapted Cochrane Risk of Bias Tool [20]. mHealth interventions as well as the target populations differed between the studies. This tool was used because it gives more guidance on details for classifying the risk of bias and therefore enhances uniformity of assessment (Multimedia Appendix 3). Bias was assessed on the selection process of the study population, completeness of data (example number of dropouts), origin of the data (measurements performed by authors or database research), blinding of the researchers or clinicians, the presence of a clear definition of the outcomes that were used, and whether confounders were taken into account in analysis. Risk of bias was assigned as either low risk, high risk, or unclear risk. The quality assessment tool can be found in Multimedia Appendix 3. Validity of the papers was taken into account in the Discussion section.

### Synthesis of Results

Studies were grouped into 2 types: intervention and descriptive. Intervention studies employed more rigorous nonrandomized study designs used for evaluating interventions [21], whereas descriptive studies used mainly cross-sectional designs or were case studies. Data synthesis aims to give a narrative analysis. First, an overview of the scope of mHealth interventions is provided. The scope of the studies consists of the year of publication, region of the world, form of mHealth intervention, the mHealth function, topic addressed, and target population.

Narrative synthesis of the intervention studies are presented in an evidence table, in which the studies are analyzed according to their year of publication, study design, location or setting, target population, whether evaluation measures are maternal or neonatal, form of mHealth, mHealth functions related to data collection, educational, and communication and finally relevant findings. A similar evidence table was used to summarize the findings of the descriptive studies. Heterogeneous outcomes, settings, and varying study designs limited our ability to group the results of 2 or more papers together to conduct a meta-analysis for an overall quantitative conclusion. A strengths, weaknesses, opportunities, and threat analysis was also performed for all the included studies, as well as for mHealth as an intervention.

## Results

### Overview of Included Studies

**Figure 1:**
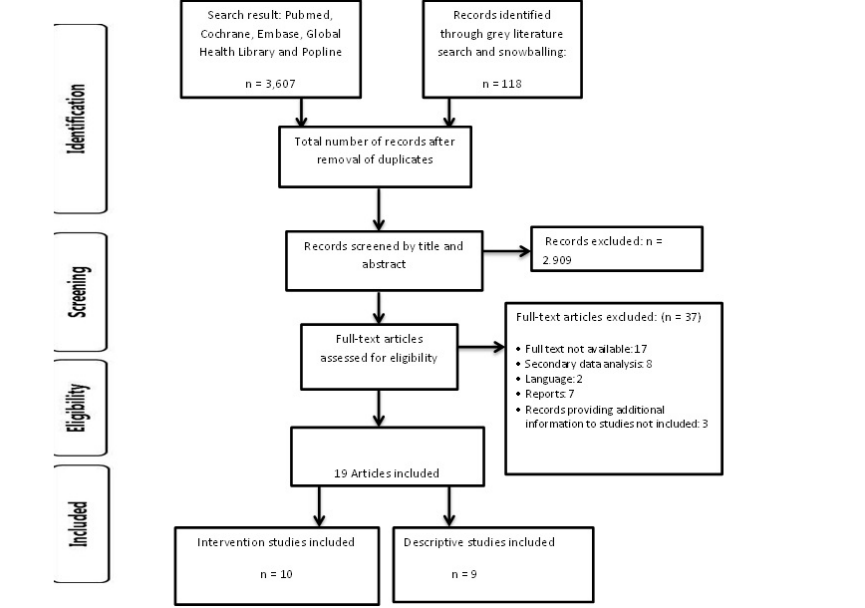
PRISMA flow diagram of studies included in this review.

A total of 3725 papers were identified in the database and grey literature searches. After removal of duplicates using Endnote (version 11), 2965 articles remained and were screened by title and abstract. This resulted in exclusion of 2909 articles, leaving 56 articles to screen for eligibility. Thirty-seven articles were further excluded. Reasons for exclusion included unavailability of full text (n=17), language (n=2), secondary analysis (n=8), reports (n=7), and unavailability of records providing additional but key information on studies (n=3). A total of 19 articles were included in our study, 10 intervention studies and 9 descriptive studies. Figure 1 illustrates the study screening and selection process, whereas Table 1 provides an overview of the scope of mHealth interventions in the included studies. Overall, 73.7% (14 of 19) of the studies were conducted in Africa and 26.3% (5 of 19) in Asia. Most interventions (68.4%) used SMS text messaging, but the form of text messaging varied between the studies. Text messaging forms included unidirectional and multidirectional text messages. Furthermore, 10.5% (2 of 19) of studies combined SMS text messages with another form of mHealth. mHealth function as well as target populations differed between the studies.

Regarding the quality of mHealth evaluations in the studies, only one of the intervention studies was a randomized controlled trial (RCT) [22]. The others [23-31] had quasi-experimental nonrandomized designs. Table 2 provides an overview of the characteristics of the intervention studies, respectively by RCT and nonrandomized controlled trial status. Study designs reported for the descriptive studies ranged from case studies to cross-sectional study designs [32-40]. Table 3 provides an overview of the characteristics of the descriptive studies and their key findings.

**Table 1:** Scope of studies included in the review.

Category	Subcategory	Intervention studies (N=10)		Descriptive studies (N=9)	
		Number of studies	% of studies	Number of studies	% of studies
**Region**	Africa	8	80.0	6	66.7
	Asia	2	20.0	3	33.3
**Form of mHealth**	Unidirectional text messaging	3	30.0	3	33.3
	Multidirectional text messaging	2	20.0	1	11.1
	Multidirectional text and voice messages	1	10.0	1	11.1
	Unidirectional text messaging and Web-based technology	1	10.0	1	11.1
	Mobile phone health apps or surveys	2	20.0	2	22.2
	Mobile phone software	-	-	1	11.1
	Mobile phone recording	1	10.0	-	-
**mHealth function (certain studies contain multiple forms)**	Data collection	6	60.0	4	44.4
	Educational	1	10.0	3	33.3
	Communication or information sharing	5	50.0	2	22.2
**Topic (certain studies contain multiple topics)**	Postpartum hemorrhage	1	10.0		
	Skilled maternal and newborn care	4	40.0	2	22.2
	Training or educating midwives and nurses	2	20.0	4	44.4
	Reproductive health	1	10.0	-	-
	HIV and pregnancy	2	20.0	1	11.1
	Malaria in pregnancy	-	-	1	11.1
	Postnatal depression	-	-	1	11.1
	Infant feeding	1	10.0	-	-
**Target population (certain studies target multiple populations)**	Traditional birth attendants	2	20.0	1	11.1
	Health extension workers	1	10.0	1	11.1
	Midwives	2	20.0	2	22.2
	Health care staff	2	20.0	2	22.2
	Medical students	1	10.0	-	-
	Community health workers	3	30.0	4	44.4
	Health surveillance assistants	1	10.0	-	-

### Overall Risk of Bias Assessment of Intervention Studies

**Figure 2:**
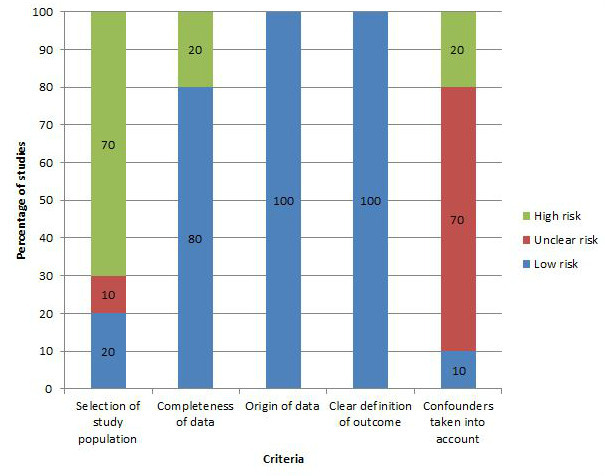
Graphical presentation of risk of bias assessment for intervention studies included in the review.

The overall risk of bias assessment is reported in Figure 2 and Multimedia Appendix 4. All included studies provided a clear definition of the outcomes and made use of self-measured data. Only one study accounted for confounders in the analysis [27], 2 studies did not [24,29], and the remaining 8 of the included studies did not mention whether or not this was the case [22,23,25,26,28,30,31,40]. Most studies had a high risk of bias for selection of study population, with one study with unclear risk of bias [25]. Two studies scored a high-risk bias for completeness of data, one study did not make use of a control group [24], and another paper performed the study without secondary monitoring [23].

### Narrative Synthesis of Results of Intervention Studies

The intervention studies distinguish the use of mHealth directed at health care providers for data collection, communication, or educational purposes.

### mHealth as a Data Collection Tool

Six papers described an mHealth intervention used as a data collection tool [22-24,26-28].

Two of these studies assessed the knowledge and skill retention of midwives and TBAs after training sessions on how to use mobile phones as data collection tool [27,28]. In rural Liberia, TBAs were trained in SMS texting data collection protocol and performed a 1-year posttest evaluation that showed that 63.6% of them displayed evidence of statistically significant knowledge and skill retention [28]. However, only 11.0% of the TBAs were able to perform more complex tasks, such as adding credit to the mobile phone to increase mobile phone usage time. This task proved to be difficult due to low literacy and poor mobile phone reception and 69.8% of TBAs relied on people with higher education to support them with mobile functions. In rural Liberia, traditional midwives were trained to use SMS texting for real-time remote data collection using a pregnancy reporting protocol. They showed a significant mean increase in mobile phone knowledge and skill acquisition afterward [27].

In a study conducted in Ethiopia, most health workers were able to use mobile phone health apps that are appropriate for their technical needs in terms of maternal health data collection [26], suggesting that mobile technologies allow health managers to more quickly and reliably have access to data which can help them identify whether and where there are issues in service delivery. Evaluation of mobile phone use by TBAs to report incidence, management, and outcome of postpartum hemorrhage, showed that 90.0% of the TBAs used their mobile phones to send SMS text messages in the 90 days following training and were able to use the protocols to report clinical outcomes [23]. In 4 Chinese villages, the use of mobile phone questionnaire for data collection on infant feeding practices was compared with a pen-and-paper method. The pen-and-paper method was observed to be more error prone [22] as 65.0% of the pen-and-paper records did not match and needed additional checking. Cost-wise, however, the mobile phone method was considerably more expensive at US $23 per questionnaire, compared with the pen-and-paper method that cost US $13 per questionnaire. In a study in Myanmar, a combination of Web-based and mobile technology was used to generate antenatal care and expanded program on immunization visit dates, in which the health worker was able to cross-check, identify, and update the mother and child status at the health facility or during home visits [24]. This tool improved antenatal care and expanded program on immunization coverage in the study area with less delay in antenatal and immunization visits.

### mHealth as Communication Tool

Three studies assessed the use of mHealth interventions as a communication tool [25,29,30]. In Malawi, it was shown that mobile phones can be used to reduce the communication gap between health workers and their district teams [25]. The intervention consisted of SMS text messages used to report stock-outs, asking for general information, reporting emergencies, confirming meetings, and requesting technical support. For CHWs in the intervention group, an average of 9 minutes and at an average cost of $0.6 to report issues and receive feedback per contact was estimated. The most common modes of communication among this group were phone calls (94.4%) and SMS text messages (100.0%). Face-to-face communication was only reported by 8.0% of the participants. For CHWs in the control district, 1681 minutes (28 hours) and an average cost of US $4.6 to report and receive feedback per contact were estimated. The most common modes of communication amongst this group were use of face-to-face communication with their supervisors at district level (92.0%) and phone calls (6.0%) with none of them using SMS text messages. The mHealth intervention was at least 4 times cheaper and 134 times more efficient, compared with traditional and most common methods of walking, biking, or using public transport to reach supervisors face to face.

SMS texting between CHWs and either ambulance, health facility staff, district hospital, and central level, enabled an effective and real-time 2-way communication alert system to reduce maternal and child health deaths in Rwanda [29]. The mHealth intervention resulted in a 20.0% increase in facility-based delivery, from 72.0% to 92.0%. In addition, CHWs became more proactive in identifying pregnant women and following up on registered pregnant women for appropriate care, by sending reminders to their mobile phones. In a study conducted in Zambia, SMS text messaging was used for transmission of test results between health facilities and caregivers to reduce the time needed for diagnosis of infant HIV infection [31]. The mean turnaround time for delivery of a test result to the relevant health facility decreased from 44.2 days preimplementation to 26.7 days postimplementation. The mean turnaround time for delivery of test results to the caregiver of the tested child decreased from 66.8 days to 35 days [31].

**Table 2:** Characteristics of the intervention studies included in the review and the relevant findings.

Study/ (Focus of evaluation)	Study design	Target population or size (Setting)	Form of mHealth	mHealth function	Relevant findings
2014, Munro et al [28] (Maternal)	Nonrandomized design (preintervention and postintervention evaluation)	99 TBAs^a^ trained; 63 retained 1-year posttraining for complete evaluation (Rural Bong county in Liberia)	Utilization of mobile phone functions, coded SMS text messaging	Data collection and transmission (locating pregnant women; take data on age and referring them for antenatal care)	Participants demonstrated an increase in the mean number of skills that they were able to perform between pretest and both the immediate posttest and 1-year posttest. Mean number of skills that the participants were able to complete did decrease slightly between immediate and posttest. Individual skills verified a significant retention of knowledge between the pretest and posttest. Many TBAs continued to have trouble with the more complex skills of adding credit to a mobile phone (11% was able to do this at posttest). 70% of TBAs relied primarily on others with higher education to assist them with mobile functions; 24% used their phone to communicate with the certified midwife; 14.3 % wanted to communicate, but had poor reception.
2014, Pathfinder (Grey Literature) [30] (Maternal)	Nonrandomized design (preintervention and postintervention evaluation)	258 participants in an infant feeding health education program (Abuja and Nasawara in Nigeria)	150 CHW^c^ in 10 primary health clinics	Education and communication (ANC^d^ protocols, and client follow-up)	CHWs increased HIV^e^ testing from 68% to 82%. Blood pressure measurement increased from 87% to 97%. The quality of care score from client interviews increased from 13.33 at baseline to 17.15 at end line, with the most significant improvements related to health counseling. Individual and group health counseling sessions became structured.
2013, Little et al [26] (Maternal)	Nonrandomized design (preintervention and postintervention evaluation	20 HEW^f^, 12 midwives, 5 supervisors (Kilte and Awelalo districts in the Tigray region of Ethiopia)	Mobile phone app using open source components	Data collection (using appropriate technologies to meet needs of HEW and midwives	GPRS^g^ connection was available in 35 health posts and centers (74%) of the study districts. There were very few instances of the mobile data network being unavailable for a substantial period of time. 34 of the 36 phones were retained; 2 were stolen with one later recovered; 3 phones had issues with insensitive screens and were replaced. Most health workers rapidly learned how to use and became comfortable with the touch screen devices so only limited technical support was needed.
2012, Zhang et al [22] (Maternal and Neonatal)	Randomized controlled trial	10 students of the Hebei Union School of Public Health (Zhaozhou Township, Hebei Province, China)	Mobile phone data collection	Data collection (Use of mobile phones for data collection on infant feeding practices compared with use of pen and paper)	In 120 copies of pen-and-paper questionnaires, 55 questionnaires contained errors. 65% of the pen-and-paper records did not match and needed to be checked. There was no significant difference between duration of pen-and-paper method versus mobile phone method. The mean cost per questionnaire was higher for the mobile phone questionnaire (US $23) than for the pen-and-paper questionnaire (US $13). The mobile phone method was acceptable to interviewers, with only minor problems that did not result in data loss.
2012, Lori et al [27] (Maternal)	Nonrandomized design (preintervention and postintervention evaluation)	99 TBAs (Rural Liberia)	SMS text messaging	Data collection (using a pregnancy reporting protocol)	Mean increase in mobile phone knowledge scores was 3.67 (95% CI 3.39-3.95). Data collectors also demonstrated a significant increase in their ability to perform each individual mobile phone task. Participants with a mobile phone in the family did significantly better on 3 of the 7 tasks in pretest.
2012, Seidenberg et al [31] (Infant)	Nonrandomized design (preintervention and postintervention evaluation)	At least 2 health workers from each facility (2 districts in Southern Zambia)	SMS text messaging	Data collection and transmission (to reduce the time between blood sampling for the detection of infant HIV infection and notification of the test results to the relevant point-of-care health facility by using SMS-based system	Mean turnaround time for delivery of a test result to the relevant health facility fell from 44.2 days (SD^h^:28) preimplementation to 26.7 days (SD:31.8) postimplementation. Mean turnaround times for delivery of a test result to a caregiver of the tested infant were 66.8 (SD: 38.8) preimplementation and 35 days (SD: 31.2) postimplementation.
2012, Lemay et al [25] (Maternal)	Nonrandomized controlled trial (staged design)	Health surveillance assistants and community health workers. 95 SMS users in Salima. 95 nonusers in Salima. 95 nonusers in Kasungu (Salima, Nkhotakota, and Kasungu Districts in Malawi)	SMS text messaging	Communication (reducing communication gaps between health workers and their district teams; increasing access to information and improve quality of services)	SMS used to report stock-outs, asking general information, reporting emergencies, confirming meetings, and requesting technical support. Among respondents who received phones, the most common form of communication was SMS (100%), phone calls (94%), public transport to travel (8%). Among respondents who did not receive phones: 92% used transport and only 6% used phone calls. None used SMS for communication. SMS participants needed an average of 9 minutes to report issues and receive feedback at an average of USD 0.61$. Health workers with no access to SMS spend an average of 1445 minutes (24 hours) to report and receive feedback at an average of USD 2.70$. In control district, it took 1681 minutes to report and receive feedback at an average of USD 4.56$.
2012, Ngabo et al [29] (Maternal)	Nonrandomized design (pre intervention and postintervention evaluation)	432 community health workers and the rest of the health system (Musanze, Rwanda)	SMS Text messaging (Rapid SMS-MCH^i^ system)	Communication (SMS-based platform, enabling effective and real time 2-way communication for action, between CHWs at community level, and the rest of the health system. Used to improve access to antenatal, postnatal care, institutional delivery, and emergency obstetric care)	5734 SMS were sent. 11,502 pregnancies (81% of the 14,200 estimated annual pregnancies in district) were monitored. A 20% increase in facility-based delivery from 72% 12 months before to 92% at the end of pilot phase.
2011, Andreatta et al [23] (Maternal)	Nonrandomized design (posttraining evaluation)	8 TBA, 2 professional nurse midwives (Sene District in Ghana)	SMS text messaging	Data collection and communication (reporting postpartum hemorrhage occurrence, management, and outcome)	Both professionals and TBAs were able to use the specified reporting and text messaging protocols to report clinical outcomes. 425 births were reported during study period, with 13 cases of PPH^j^occurring (3.1%) cases.
2010, Kwaewkungwal et al [24] (Maternal and child, including neonatal)	Nonrandomized design (preintervention and postintervention evaluation)	Health workers in charge of ANC or EPI^k^ services (sample size not indicated in paper) (Phung district, Thai-Myanmar)	SMS text messaging and Web-based apps	Data collection, automated generation of list, and update information regarding the antenatal care and child's immunization status on mobile phone when performing ANC or EPI activities off health care clinic	59% come on time as per scheduled dates after implementation compared with 44% before implementation. 44% of children who came to receive scheduled vaccines on time on the preset monthly immunization date after intervention compared with 35% before. Updating immunization data on mobile phone increased odds of EPI on time by 2.04.

^a^TBA: traditional birth attendant.

^b^SMS: short message service.

^c^CHW: community health worker.

^d^ANC: antenatal care visit.

^e^HIV: human immunodeficiency virus.

^f^HEW: health extension worker.

^g^GPRS: general packet radio service.

^h^SD: standard deviation.

^i^MCH: maternal and child health.

^j^PPH: postpartum hemorrhage.

^k^EPI: expanded program on immunization.

### Narrative Synthesis of Results of the Descriptive Studies

In Indonesia, a theoretical model on the use of mobile phones to enhance the capacity of health workers was developed and tested among 223 midwives [35]. Mobile phone use was positively associated with midwives’ access to institutional and peer information resources. SMS text messaging was used to educate midwives in a study conducted in South Africa and improved clinical practice was reported by 72.0% of participants. More than two-thirds (68.0%) of the midwives commonly shared and discussed the messages with their colleagues. All participants requested to receive more text messages on other important topics [40]. Again, in rural South Africa, the feasibility and acceptability of using mobile phone app for data collection amongst pregnant women living with HIV was explored [38]. Acceptability was high, as well as perceived usefulness and ease of use. Feasibility of conducting the interviews in the setting was also high, with no significant challenges with respect to network coverage, cost of hardware and software, and secure transmission of data. Among nurses receiving midwifery education, mobile phone usage facilitated authentic problem solving, reflective practice, and life-long learning [36]. In Afghanistan, World Vision rolled out an mHealth intervention, using the open source CommCare platform [39]. This was aimed at improving the quality of counseling for pregnant women, promote facility delivery, and facilitate timely referral of women and newborns to facilities and hopefully result in decreased maternal and newborn deaths following increase in utilization of services [39]. The study does not report on how these were achieved but mentions that it was successful, and the project was further expanded to deal with childhood nutrition. In one study, 9 health workers were provided with mobile phones with an installed algorithm that allowed for real-time access to data. This reduced time lag in patient data transmission and allowed for pregnant women to be categorized based on risk for treatment [32]. The Safer Deliveries project in Zanzibar, which employed an open source mobile app enrolled TBAs and resulted in an increase in registration of pregnant women as well as increase of facility delivery from 33.6% to 71.0% [33]. Finally, the International Institute for Communication and Development in 2013 piloted a project in Mali in which CHWs and specialists used a mobile app to report and monitor cases of malaria in pregnancy and among children aged younger than 5 years, as well as detect and respond promptly to any outbreak [34]. This resulted in a 31% reduction in malaria in pregnancy and 33% reduction in children under 5 years with malaria. Table 3 presents an overview of the characteristics and relevant findings of the descriptive studies.

### Strengths, Weaknesses, Opportunities, and Threats Analysis of Included Studies

A Strengths, Weaknesses, Opportunities, and Threat analysis was conducted for all included studies. All studies included are relatively current studies published from 2010 onward providing up-to-date information. There is also a good variation of settings within the domain of LMIC, with studies conducted in West, East, and Southern Africa, and different parts of Asia. All forms of mHealth interventions as well as different functions that can be served by mHealth are represented in these studies. In all but one study, standardized phones were procured for participants. Strength of the included studies is the broad range of health worker categories considered, allowing for easy assessment of feasibility of mHealth apps for the daily work of health care workers. Weaknesses in the studies related mainly to their study design. Only one of the intervention studies was a RCT [22], the others combined various nonrandomized study designs, such as preintervention and postintervention comparison, and in this regard, lack the specific rigor associated with intervention studies. The descriptive studies ranged from surveys to case studies. For each broad mHealth function, various apps were reported. For example, under communication function, one study reported mHealth used to report stock-outs to higher levels [25], whereas another study reported mHealth used to follow-up women and report clinical outcomes [23]. This could have been an advantage if more than one study reported specific apps to allow for comparison of results. Most of the studies report on process rather than outcome measures, and thus, this systematic provides an indication for opportunity for more rigorous intervention studies that focus on both specific maternal and neonatal outcome measures in the future.

### Strengths, Weaknesses, Opportunities, and Threats Analysis of mHealth as an Intervention

Multiple studies mention low costs to be strength of the mHealth interventions compared with traditional methods [24,25,31,40]. mHealth interventions were found to be considerably more efficient than traditional methods used for communication [25], and to improve the effectiveness of community health services in terms of managing logistics, reporting events, and addressing emergencies. mHealth allowed for integration of all levels of health workers, including TBAs, to expedite emergency referrals and communicate with skilled providers like midwives [28].

Weaknesses included that the information in the text messages was too simple and needed additional detailed information [40]. In addition, remoteness of study sites was a limitation to Web-based education due to lack of or poor access to the Web [40]. Technological problems such as poor reception, lost and damaged phones, and difficulty with certain mobile phone models were also identified [40]. mHealth interventions that are dependent on existing information systems, with modular systems that are not interoperable, cannot be linked to other settings and data structures [24]. Other weaknesses include poor telephone maintenance and lack of or limited access to electricity in a number of communities.

Clear opportunities exist for utilization of mHealth. This includes the additional functions of the technology, such as global positioning system, taking and storing pictures and videos, as well as the ability to record sound. These can facilitate data collection tools in the future [29]. Another opportunity offered by mHealth is for a broader mobile network coverage that could expand the reach of health information to frontline health workers in remote areas and accelerate knowledge exchange between health workers and higher levels in the health system [25]. mHealth offers the opportunity for strengthening of health care infrastructure with the requisite financial support, and the technology can be applied to a broader scope of public health care [27]. It is also possible to address various health system issues using one mHealth program or intervention [37,39], and there are various software available that could be adapted to suit specific needs of health care [34]. Opportunities for public–private partnership also exist [29].

Factors that threaten mHealth implementation included lack of reliable Web coverage, which limits the potential of mHealth in the public sector [28,40], limited capacity to manage damaged phones, low literacy levels [28], and lack of appreciation by health workers of the need to use data where it is generated [24]. Several of the reported interventions were conducted by foreign agencies that could potentially result in limited sustainability [30,33,34,39]. This, however, could be overcome if local stakeholders and institutions engaged in the program appreciate the value of the capacity building offered. Finally, it is important that any tool adapted to be used through mHealth be itself efficient, to maximize the benefits of mHealth functions [37].

**Table 3:** Characteristics of the descriptive studies included in the review and the relevant findings.

Study (Focus of Evaluation Measures)	Study design	Target health workers or size (Setting)	Form of mHealth	mHealth function	Relevant findings
2014, Lee et al [35] Maternal and Neonatal)	Cross-sectional	223 midwives (15 sub-districts of Aceh Besar, Indonesia)	One-way mobile phone use	Improving access to health-related resources: formal (medical professionals) and informal (peer workers) resources	Mobile phone use was positively associated with midwives' access to institutional and peer information resources. Access to institutional resources was positively associated with midwives' health knowledge, whereas access to peer resources was not. Access to peer resources was associated with higher self-efficacy, which was positively associated with health knowledge. Implications for technology interventions strategies targeted to community health workers in rural communities provided.
2014, Tsai et al [37] (Maternal)	2 Cross-sectional studies	Community health workers (sample size not stated in paper) (Khayelitsha Cape Town, South Africa)	Mobile phone program	Case finding (use of mobile phones for administering the EPDS^a^ during the routine course of their community-based outreach and wellness work	CHWs^b^ were able to detect probable antenatal depression using the scale during their routine outreaches with excellent discrimination, with area under the receiver-operating characteristic curve (AUC) values ranging from 0.91 to 0.99; 0.97 sensitivity and 0.76 specificity.
2014, Pimmer et al [36] (Maternal)	Case study	16 nurses attending an advanced midwifery course (Rural South Africa)	Mobile phones	Nurse education (mobile phones as educational tools)	Nursing students in resource poor settings use mobile technology as educational tools. These learning practices involve sociocognitive processes, learning in the form of joint problem solving and reflection, as well as more intensive forms of sociocultural participation. In order for educational institutions to more fully and more systematically harness the potentials of these media, a number of ethical and practical issues need to be addressed.
2014, D-Tree International [33] (Grey Literature) (Maternal and neonatal)	Cross-sectional	24 TBAs in phase I and 223 CHWs in phase II (Zanzibar, Tanzania)	Mobile phone with open source mobile app	Data collection and communication and information sharing	There was an increase in access to skilled care during pregnancy, childbirth, and post-partum care. 71% facility delivery compared with 33.6% in DHS.^c^ 77% facility delivery in phase II (DHS range between 25% and 41%). Increase in primary care level deliveries (44% compared with 4% in 2011). Geographical differences in delivery habits highlighted. Increased self-efficacy and capability of frontline workers.
2014, World Vision [39] (Maternal and neonatal)	Cross-sectional	CHWs (sample size not provided in paper) (Afghanistan)	Mobile phone	Counseling and referrals	Promotion of health facility deliveries. Timely referrals of women and newborns. Decrease in maternal and new-born deaths (worth noting that exact measures of these are not stated in the paper).
2013, van Heerden et al [38] (Maternal)	Cross-sectional	16 data collectors; (Rural South Africa)	MPAPI^d^	Data collection (feasibility of face-to-face maternal health data collection from pregnant women living with HIV using a mobile phone survey app)	Perceived usefulness was reported to be slightly higher than perceived ease of use. After 3 months of field use, interviewer perceptions of both perceived ease of use and perceived usefulness were found to be higher than before training. High feasibility of conducting MPAPI interviews in this setting. Network coverage was available in all clinics and hardware, software, cost, and secure transmission to the data center presented no significant challenges over the 21-month period. For the 12 MLH^f^participants in group 2, anxiety about the multimedia capabilities of the phone was evident. Their concern centered on the possibility that their privacy may be invaded by interviewers using the mobile phone camera to photograph them. For participants in group 3, having the interviewer sit beside versus across from the interviewee during the MPAPI interview was received positively by 95% of MLH. Privacy (6%) and confidentiality (5%) concerns were low for group 3 MLH. Mobile phones were found both to be acceptable and feasible in the collection of maternal and child health data from women living with HIV in South Africa.
2013, IICD^g^ [34] (Grey Literature) (Maternal and children)	Cross-sectional	50 CHWs and 10 health specialists (Yirimodjo, Mali)	MAMMA^h^	Data collection and monitoring (questionnaire with malaria indicators and monitoring of disease evolution)	31% reduction in malaria in pregnancy cases. Faster treatment response (65%). 32% reduction in malaria in children under 5 years. 20% increase in pregnant women sleeping under bed net. 42% increase in pregnant women receiving preventative medication.
2012, Woods et al [40] (Maternal and neonatal)	Cross-sectional	50 midwives out of 2500 midwives from public and private sectors (South Africa)	SMS text messaging; with link to a website with additional information	Education	86% enjoyed and learned from weekly text messages. 72% felt that the messages improved clinical practice. 68% shared and discussed the messages with colleagues.
2010, Alam et al [32] (Grey Literature) (Neonatal)	Case study	9 BRAC^i^ health workers (3 urban slums of Dhaka, Bangladesh)	Mobile phones with smart algorithms (The Click Module)	Data collection (real-time access to data)	Health workers could send data directly to the central MIS^j^ system. Reduced time lag in data transmission. Established a secured Web page containing all patient data. Established an automated decision tree that categorizes patients depending on their risk levels.

^a^EPDS: Edinburgh Postnatal Depression Scale.

^b^CHWs: community health workers.

^c^DHS: demographic and health survey.

^d^MPAPI: mobile phone-assisted personal interviewing.

^e^HIV: human immune deficiency virus.

^f^MLH: mothers living with HIV.

^g^IICD: International Institute for Communication and Development.

^h^MAMMA: Mamans Mobiles contre le Malaria au Mali.

^i^BRAC: building resources across community (a nongovernmental organization).

^j^MIS: management information system.

## Discussion

### Principal Findings

This systematic review shows effective use of mHealth interventions as communication, educational, and data collection tools by health workers to report on medical events related to maternal and child health within their community. These constitute health systems strengthening app tools [41]. The specific mHealth functions have enabled health workers to track pregnant women in their care, as well as facilitate referrals. Such strategies, targeted at data collection and reminders for antenatal visits, directly impact on service utilization such as antenatal coverage, whereas those aimed at improving skilled attendance at delivery and facility delivery impact more directly on mortality [42]. Unfortunately, only one of the studies included in our review directly reports the effect of mHealth on maternal and neonatal mortality, without exact details of coverages. Although other studies have shown that mHealth interventions targeted at clients can reduce perinatal mortality by 50% [43], we could not convincingly demonstrate this effect for health worker-targeted interventions.

The fact that most of the studies included in this review targeted health workers at community level provides insights into the possibility of creating an intermediate layer in which health workers form an important linkage between higher health institutions and the community in harnessing the befits of mHealth.

Some challenges of mHealth were identified, and these were mainly technological problems, such as mobile network coverage, Web-based access, electricity access, and maintenance of mobile phones [11,29,40]. These could negatively affect the expansion of the mHealth interventions in LMIC if not addressed. However, technological improvement comes with associated costs. Engaging the private sector in a public-private partnership can reduce such cost [29] and facilitate the expansion of mHealth interventions in LMIC in the future. Decreasing costs of handsets will potentially reduce further the cost of mHealth interventions.

Given the large investments in mHealth [44,45], with the highest cost of service provision in LMIC [6], experimental evaluations which will thoroughly assess its impact [45], more specifically on maternal and neonatal health outcomes, will be most beneficial. Future studies should investigate the effectiveness of the interventions by measuring similar outcomes. Health workers’ level of literacy affected their ability to perform complex tasks on mobile devices [27]. It is important to address this challenge if the full complement, respectively potential of mHealth is to be deployed at all levels of service delivery, including the community level. Lower cadre of health staff will need adequate training to ensure their optimization of such interventions.

### Limitations

Limitations of our review include a high risk of bias observed for some of the intervention studies, mainly relating to limited consideration of confounding. Only one study was a RCT. Most studies were pilot or implementation studies. A further limitation of the current systematic review is the domain limitation of LMIC. This affects the generalizability of our results to other settings, as we are aware that some studies in high-income countries or low-income mothers in high-income countries could provide informative insight in the effectiveness of mHealth interventions to improve health outcomes [13].

The strength of the current systematic review is the comprehensive search conducted including available grey literature reflecting current activities of nongovernmental organizations, which are often not published in peer-reviewed journals. This paper thus provides a comprehensive overview of the available literature on the effectiveness of mHealth interventions to date and narratively assesses the broad function of mHealth. The methodology used in the narrative synthesis looks at the broad function of mHealth as used in the study, the targeted frontline providers, and the effectiveness of the mHealth intervention, an approach that facilitated easy assessment of the usefulness of the various mHealth functions.

### Conclusions

This systematic review indicates that mHealth interventions targeting health care workers have the potential to materially improve maternal and neonatal health services in LMICs. There is, however, a gap in the knowledge of how mHealth interventions directly affect maternal and neonatal outcomes and future research should employ experimental designs to address this gap.

## Appendix

Multimedia Appendix 1 List of organizations contacted for grey literature.

Multimedia Appendix 2 Full search strategy.

Multimedia Appendix 3 Adapted quality assessment tool.

Multimedia Appendix 4 Table showing Risk of Bias assessment for included intervention studies.

## Abbreviations

CHW: community health worker
HIV: human immunodeficiency virus
LMIC: low- and middle-income country
RCT: randomized controlled trial
SMS: short messaging service
TBA: traditional birth attendant
