# Supplementary material for: Effectiveness of mHealth Interventions Targeting Health Care Workers to Improve Pregnancy Outcomes in Low- and Middle-Income Countries: A Systematic Review
Source: J Med Internet Res. 2016 Aug 19;18(8):e226. doi: 10.2196/jmir.5533 (PMC5010646; doi:10.2196/jmir.5533)
Supplement: Multimedia Appendix 1 [file jmir_v18i8e226_app1.pdf]

Supplement 1: List of Organizations

Aga Khan Health Service, Tanzania, AMREF Health Africa, The BRAC MNCH (Maternal, Neonatal and Child Health) programme, Cell Life Project, Concern Worldwide, D-Tree Internataional, The Innovation Working Group, Family Health International (FHI), Grameen Foundation, GSMA for Mobile Development, International Institute for Communication and Development (IICD), Johns Hopkins (JHPIEGO), Royal Tropical Institute (KIT), Mobile Alliance for Maternal Action (MAMA), Marie Stopes, Medic Mobile, mHealth Alliance, Mothers2Mothers, NEEDS, Pathfinder, Philani, Pharmaccess International, PLAN International, Red Cross, USAID, UNICEF, Foundation for Innovative New Diagnostics, World Health Organization, Women Deliver, World Bank, World Vision International, INTRAHEALTH, Afya Research Africa, Click Diagnostics, EngenderHealth, Village Reach, Wired Mothers, Simavi, African Women Foundation, CDC,
