# Supplementary material for: Effectiveness of mHealth Interventions Targeting Health Care Workers to Improve Pregnancy Outcomes in Low- and Middle-Income Countries: A Systematic Review
Source: J Med Internet Res. 2016 Aug 19;18(8):e226. doi: 10.2196/jmir.5533 (PMC5010646; doi:10.2196/jmir.5533)
Supplement: Multimedia Appendix 2 [file jmir_v18i8e226_app2.pdf]

Supplement 2: Full search strategy

1.1 Search Pubmed/MEDLINE (25 July 2014):
((#1 OR #2 OR (#3 AND #4) OR #5) AND #6 AND #7

#1:
(pregnancy[Title/Abstract] OR mother*[Title/Abstract] OR women, pregnant[MeSH Terms] OR woman, pregnant[MeSH Terms])

#2:
(midwife[Title/Abstract] OR midwives[Title/Abstract] OR lay midwife[Title/Abstract] OR lay midwives[Title/Abstract] OR traditional midwife[Title/Abstract] OR traditional midwives[Title/Abstract] OR nurse midwife[Title/Abstract] OR nurse midwives[Title/Abstract] OR birth attendant*[Title/Abstract] OR traditional birth attendant*[Title/Abstract] OR obstetrician*[Title/Abstract] OR gynaecologist*[Title/Abstract] OR gynecologist*[Title/Abstract] OR nurse midwives[MeSH Terms] OR midwifery[MeSH Terms])

#3:
(health care provider*[Title/Abstract] OR community health worker*[Title/Abstract] OR nurse*[Title/Abstract] OR nursing personnel[Title/Abstract] OR nursing staff[Title/Abstract] OR medical doctor*[Title/Abstract] OR doctor*[Title/Abstract] OR clinical officer*[Title/Abstract] OR COH[Title/Abstract] OR medical officer*[Title/Abstract] OR MOH*[Title/Abstract] OR health personnel[Title/Abstract] OR health staff[Title/Abstract] OR health personnel[MeSH Terms] OR community health workers[MeSH Terms] OR community health worker[MeSH Terms] OR nurses[MeSH Terms] OR nurses, public health[MeSH Terms])

#4:
(maternal[Title/Abstract] OR newborn*[Title/Abstract] OR antenatal[Title/Abstract] OR obstetric[Title/Abstract] OR postnatal[Title/Abstract] OR postpartum[Title/Abstract] OR prenatal[Title/Abstract] OR perinatal[Title/Abstract] OR infant*[Title/Abstract] OR interpartum[Title/Abstract] OR neonatal[Title/Abstract] OR baby[Title/Abstract] OR babies[Title/Abstract])

#5:
(maternal[Title/Abstract] OR newborn*[Title/Abstract] OR antenatal[Title/Abstract] OR obstetric[Title/Abstract] OR postnatal[Title/Abstract] OR postpartum[Title/Abstract] OR prenatal[Title/Abstract] OR perinatal[Title/Abstract] OR infant*[Title/Abstract] OR interpartum[Title/Abstract] OR neonatal[Title/Abstract] OR baby[Title/Abstract] OR babies[Title/Abstract] OR maternal child nursing[MeSH Terms] OR maternal health services[MeSH Terms] OR delivery, obstetric[MeSH Terms] OR obstetrics[MeSH Terms] OR postnatal care[MeSH Terms] OR prenatal care[MeSH Terms] OR perinatal care[MeSH Terms] OR neonatal nursing[MeSH Terms])

#6:
(((((low resource[Title/Abstract]) OR limited resource[Title/Abstract]) OR constrained resource[Title/Abstract]) OR restricted resource[Title/Abstract]) OR delimited resource[Title/Abstract]) OR restrained resource[Title/Abstract]) OR emerging country[Title/Abstract]) OR emerging countries[Title/Abstract])) OR (Africa[Title/Abstract] OR Asia[Title/Abstract] OR Caribbean[Title/Abstract] OR West Indies[Title/Abstract] OR South America[Title/Abstract] OR Latin America[Title/Abstract] OR Central America[Title/Abstract] OR Afghanistan[Title/Abstract] OR Albania[Title/Abstract] OR Algeria[Title/Abstract] OR Angola[Title/Abstract] OR Antigua[Title/Abstract] OR Barbuda[Title/Abstract] OR Argentina[Title/Abstract] OR Armenia[Title/Abstract] OR Armenian[Title/Abstract] OR Aruba[Title/Abstract] OR Azerbaijan[Title/Abstract] OR Bahrain[Title/Abstract] OR Bangladesh[Title/Abstract] OR Barbados[Title/Abstract] OR Benin[Title/Abstract] OR Byelarus[Title/Abstract] OR Byelorussian[Title/Abstract] OR Belarus[Title/Abstract] OR Belorussian[Title/Abstract] OR Belorussia[Title/Abstract] OR Belize[Title/Abstract] OR Bhutan[Title/Abstract] OR Bolivia[Title/Abstract] OR Bosnia[Title/Abstract] OR Herzegovina[Title/Abstract] OR Hercegovina[Title/Abstract] OR Botswana[Title/Abstract] OR Brasil[Title/Abstract] OR Brazil[Title/Abstract] OR Bulgaria[Title/Abstract] OR Burkina Faso[Title/Abstract] OR Burkina Fasso[Title/Abstract] OR Upper Volta[Title/Abstract] OR Burundi[Title/Abstract] OR Urundi[Title/Abstract] OR Cambodia[Title/Abstract] OR Khmer Republic[Title/Abstract] OR Kampuchea[Title/Abstract] OR Cameroon[Title/Abstract] OR Cameroons[Title/Abstract] OR Cameron[Title/Abstract] OR Camerons[Title/Abstract] OR Cape Verde[Title/Abstract] OR Central African Republic[Title/Abstract] OR Chad[Title/Abstract] OR Chile[Title/Abstract] OR China[Title/Abstract] OR Colombia[Title/Abstract] OR Comoros[Title/Abstract] OR Comoro Islands[Title/Abstract] OR Comores[Title/Abstract] OR Mayotte[Title/Abstract] OR Congo[Title/Abstract] OR Zaire[Title/Abstract] OR Costa Rica[Title/Abstract] OR Cote d'Ivoire[Title/Abstract] OR Ivory Coast[Title/Abstract] OR Croatia[Title/Abstract] OR Cuba[Title/Abstract] OR Cyprus[Title/Abstract] OR Czechoslovakia[Title/Abstract] OR Czech Republic[Title/Abstract] OR Slovakia[Title/Abstract] OR Slovak Republic[Title/Abstract] OR Djibouti[Title/Abstract] OR French Somaliland[Title/Abstract] OR Dominica[Title/Abstract] OR Dominican Republic[Title/Abstract] OR East Timor[Title/Abstract] OR East Timur[Title/Abstract] OR Timor Leste[Title/Abstract] OR Ecuador[Title/Abstract] OR Egypt[Title/Abstract] OR United Arab Republic[Title/Abstract] OR El Salvador[Title/Abstract] OR Eritrea[Title/Abstract] OR Estonia[Title/Abstract] OR Ethiopia[Title/Abstract] OR Fiji[Title/Abstract] OR Gabon[Title/Abstract] OR Gabonese Republic[Title/Abstract] OR Gambia[Title/Abstract] OR Gaza[Title/Abstract] OR Georgia Republic[Title/Abstract] OR Georgian Republic[Title/Abstract] OR Ghana[Title/Abstract] OR Gold Coast[Title/Abstract] OR Greece[Title/Abstract] OR Grenada[Title/Abstract] OR Guatemala[Title/Abstract] OR Guinea[Title/Abstract] OR Guam[Title/Abstract] OR Guiana[Title/Abstract] OR Guyana[Title/Abstract] OR

Haiti[Title/Abstract] OR Honduras[Title/Abstract] OR Hungary[Title/Abstract] OR India[Title/Abstract] OR Maldives[Title/Abstract] OR Indonesia[Title/Abstract] OR Iran[Title/Abstract] OR Iraq[Title/Abstract] OR Isle of Man[Title/Abstract] OR Jamaica[Title/Abstract] OR Jordan[Title/Abstract] OR Kazakhstan[Title/Abstract] OR Kazakh[Title/Abstract] OR Kenya[Title/Abstract] OR Kiribati[Title/Abstract] OR Korea[Title/Abstract] OR Kosovo[Title/Abstract] OR Kyrgyzstan[Title/Abstract] OR Kirghizia[Title/Abstract] OR Kyrgyz Republic[Title/Abstract] OR Kirghiz[Title/Abstract] OR Kirgizstan[Title/Abstract] OR ""Lao PDR""[Title/Abstract] OR Laos[Title/Abstract] OR Latvia[Title/Abstract] OR Lebanon[Title/Abstract] OR Lesotho[Title/Abstract] OR Basutoland[Title/Abstract] OR Liberia[Title/Abstract] OR Libya[Title/Abstract] OR Lithuania[Title/Abstract])) OR (Macedonia[Title/Abstract] OR Madagascar[Title/Abstract] OR Malagasy Republic[Title/Abstract] OR Malaysia[Title/Abstract] OR Malaya[Title/Abstract] OR Malay[Title/Abstract] OR Sabah[Title/Abstract] OR Sarawak[Title/Abstract] OR Malawi[Title/Abstract] OR Nyasaland[Title/Abstract] OR Mali[Title/Abstract] OR Malta[Title/Abstract] OR Marshall Islands[Title/Abstract] OR Mauritania[Title/Abstract] OR Mauritius[Title/Abstract] OR Agalega Islands[Title/Abstract] OR Mexico[Title/Abstract] OR Micronesia[Title/Abstract] OR Middle East[Title/Abstract] OR Moldova[Title/Abstract] OR Moldovia[Title/Abstract] OR Moldovian[Title/Abstract] OR Mongolia[Title/Abstract] OR Montenegro[Title/Abstract] OR Morocco[Title/Abstract] OR Ifni[Title/Abstract] OR Mozambique[Title/Abstract] OR Myanmar[Title/Abstract] OR Myanma[Title/Abstract] OR Burma[Title/Abstract] OR Namibia[Title/Abstract] OR Nepal[Title/Abstract] OR Netherlands Antilles[Title/Abstract] OR New Caledonia[Title/Abstract] OR Nicaragua[Title/Abstract] OR Niger[Title/Abstract] OR Nigeria[Title/Abstract] OR Northern Mariana Islands[Title/Abstract] OR Oman[Title/Abstract] OR Muscat[Title/Abstract] OR Pakistan[Title/Abstract] OR Palau[Title/Abstract] OR Palestine[Title/Abstract] OR Panama[Title/Abstract] OR Paraguay[Title/Abstract] OR Peru[Title/Abstract] OR Philippines[Title/Abstract] OR Philipines[Title/Abstract] OR Phillipines[Title/Abstract] OR Phillippines[Title/Abstract] OR Poland[Title/Abstract] OR Portugal[Title/Abstract] OR Puerto Rico[Title/Abstract] OR Romania[Title/Abstract] OR Rumania[Title/Abstract] OR Roumania[Title/Abstract] OR Russia[Title/Abstract] OR Russian[Title/Abstract] OR Rwanda[Title/Abstract] OR Ruanda[Title/Abstract] OR Saint Kitts[Title/Abstract] OR St Kitts[Title/Abstract] OR Nevis[Title/Abstract] OR Saint Lucia[Title/Abstract] OR St Lucia[Title/Abstract] OR Saint Vincent[Title/Abstract] OR St Vincent[Title/Abstract] OR Grenadines[Title/Abstract] OR Samoa[Title/Abstract] OR Samoan Islands[Title/Abstract] OR Navigator Island[Title/Abstract] OR Navigator Islands[Title/Abstract] OR Sao Tome[Title/Abstract] OR Saudi Arabia[Title/Abstract] OR Senegal[Title/Abstract] OR Serbia[Title/Abstract] OR Montenegro[Title/Abstract] OR Seychelles[Title/Abstract] OR Sierra Leone[Title/Abstract] OR Slovenia[Title/Abstract] OR Sri Lanka[Title/Abstract] OR Ceylon[Title/Abstract] OR Solomon Islands[Title/Abstract] OR Somalia[Title/Abstract] OR Sudan[Title/Abstract] OR Suriname[Title/Abstract] OR Surinam[Title/Abstract] OR Swaziland[Title/Abstract] OR Syria[Title/Abstract] OR Tajikistan[Title/Abstract] OR Tadzhikistan[Title/Abstract] OR Tadjikistan[Title/Abstract] OR

Tadzhik[Title/Abstract] OR Tanzania[Title/Abstract] OR Thailand[Title/Abstract] OR Togo[Title/Abstract] OR Togolese Republic[Title/Abstract] OR Tonga[Title/Abstract] OR Trinidad[Title/Abstract] OR Tobago[Title/Abstract] OR Tunisia[Title/Abstract] OR Turkey[Title/Abstract] OR Turkmenistan[Title/Abstract] OR Turkmen[Title/Abstract] OR Uganda[Title/Abstract] OR Ukraine[Title/Abstract] OR Uruguay[Title/Abstract] OR USSR[Title/Abstract] OR Soviet Union[Title/Abstract] OR Union of Soviet Socialist Republics[Title/Abstract] OR Uzbekistan[Title/Abstract] OR Uzbek OR Vanuatu[Title/Abstract] OR New Hebrides[Title/Abstract] OR Venezuela[Title/Abstract] OR Vietnam[Title/Abstract] OR Viet Nam[Title/Abstract] OR West Bank[Title/Abstract] OR Yemen[Title/Abstract] OR Yugoslavia[Title/Abstract] OR Zambia[Title/Abstract] OR Zimbabwe[Title/Abstract] OR Rhodesia[Title/Abstract])) OR (Developing Countries[Mesh:noexp] OR Africa[Mesh:noexp] OR Africa, Northern[Mesh:noexp] OR Africa South of the Sahara[Mesh:noexp] OR Africa, Central[Mesh:noexp] OR Africa, Eastern[Mesh:noexp] OR Africa, Southern[Mesh:noexp] OR Africa, Western[Mesh:noexp] OR Asia[Mesh:noexp] OR Asia, Central[Mesh:noexp] OR Asia, Southeastern[Mesh:noexp] OR Asia, Western[Mesh:noexp] OR Caribbean Region[Mesh:noexp] OR West Indies[Mesh:noexp] OR South America[Mesh:noexp] OR Latin America[Mesh:noexp] OR Central America[Mesh:noexp] OR Afghanistan[Mesh:noexp] OR Albania[Mesh:noexp] OR Algeria[Mesh:noexp] OR American Samoa[Mesh:noexp] OR Angola[Mesh:noexp] OR ""Antigua and Barbuda""[Mesh:noexp] OR Argentina[Mesh:noexp] OR Armenia[Mesh:noexp] OR Azerbaijan[Mesh:noexp] OR Bahrain[Mesh:noexp] OR Bangladesh[Mesh:noexp] OR Barbados[Mesh:noexp] OR Benin[Mesh:noexp] OR Byelarus[Mesh:noexp] OR Belize[Mesh:noexp] OR Bhutan[Mesh:noexp] OR Bolivia[Mesh:noexp] OR Bosnia-Herzegovina[Mesh:noexp] OR Botswana[Mesh:noexp] OR Brazil[Mesh:noexp] OR Bulgaria[Mesh:noexp] OR Burkina Faso[Mesh:noexp] OR Burundi[Mesh:noexp] OR Cambodia[Mesh:noexp] OR Cameroon[Mesh:noexp] OR Cape Verde[Mesh:noexp] OR Central African Republic[Mesh:noexp] OR Chad[Mesh:noexp] OR Chile[Mesh:noexp] OR China[Mesh:noexp] OR Colombia[Mesh:noexp] OR Comoros[Mesh:noexp] OR Congo[Mesh:noexp] OR Costa Rica[Mesh:noexp] OR Cote d'Ivoire[Mesh:noexp] OR Croatia[Mesh:noexp] OR Cuba[Mesh:noexp] OR Cyprus[Mesh:noexp] OR Czechoslovakia[Mesh:noexp] OR Czech Republic[Mesh:noexp] OR Slovakia[Mesh:noexp] OR Djibouti[Mesh:noexp] OR ""Democratic Republic of the Congo""[Mesh:noexp] OR Dominica[Mesh:noexp] OR Dominican Republic[Mesh:noexp] OR East Timor[Mesh:noexp] OR Ecuador[Mesh:noexp] OR Egypt[Mesh:noexp] OR El Salvador[Mesh:noexp] OR Eritrea[Mesh:noexp] OR Estonia[Mesh:noexp] OR Ethiopia[Mesh:noexp] OR Fiji[Mesh:noexp] OR Gabon[Mesh:noexp] OR Gambia[Mesh:noexp] OR ""Georgia (Republic)""[Mesh:noexp] OR Ghana[Mesh:noexp] OR Greece[Mesh:noexp] OR Grenada[Mesh:noexp] OR Guatemala[Mesh:noexp] OR Guinea[Mesh:noexp] OR Guinea-Bissau[Mesh:noexp] OR Guam[Mesh:noexp] OR Guyana[Mesh:noexp] OR Haiti[Mesh:noexp] OR Honduras[Mesh:noexp] OR Hungary[Mesh:noexp] OR India[Mesh:noexp] OR Indonesia[Mesh:noexp] OR Iran[Mesh:noexp] OR Iraq[Mesh:noexp] OR Jamaica[Mesh:noexp] OR Jordan[Mesh:noexp] OR Kazakhstan[Mesh:noexp] OR Kenya[Mesh:noexp] OR Korea[Mesh:noexp] OR Kosovo[Mesh:noexp] OR Kyrgyzstan[Mesh:noexp] OR Laos[Mesh:noexp] OR

Latvia[Mesh:noexp] OR Lebanon[Mesh:noexp] OR Lesotho[Mesh:noexp] OR Liberia[Mesh:noexp] OR Libya[Mesh:noexp] OR Lithuania[Mesh:noexp] OR Macedonia[Mesh:noexp] OR Madagascar[Mesh:noexp] OR Malaysia[Mesh:noexp] OR Malawi[Mesh:noexp] OR Mali[Mesh:noexp] OR Malta[Mesh:noexp] OR Mauritania[Mesh:noexp] OR Mauritius[Mesh:noexp] OR Mexico[Mesh:noexp] OR Micronesia[Mesh:noexp] OR Middle East[Mesh:noexp] OR Moldova[Mesh:noexp] OR Mongolia[Mesh:noexp] OR Montenegro[Mesh:noexp] OR Morocco[Mesh:noexp] OR Mozambique[Mesh:noexp] OR Myanmar[Mesh:noexp] OR Namibia[Mesh:noexp] OR Nepal[Mesh:noexp] OR Netherlands Antilles[Mesh:noexp] OR New Caledonia[Mesh:noexp] OR Nicaragua[Mesh:noexp] OR Niger[Mesh:noexp] OR Nigeria[Mesh:noexp] OR Oman[Mesh:noexp] OR Pakistan[Mesh:noexp] OR Palau[Mesh:noexp] OR Panama[Mesh:noexp] OR Papua New Guinea[Mesh:noexp] OR Paraguay[Mesh:noexp] OR Peru[Mesh:noexp] OR Philippines[Mesh:noexp] OR Poland[Mesh:noexp] OR Portugal[Mesh:noexp] OR Puerto Rico[Mesh:noexp] OR Romania[Mesh:noexp] OR Russia[Mesh:noexp] OR ""Russia (Pre-1917)""[Mesh:noexp] OR Rwanda[Mesh:noexp] OR ""Saint Kitts and Nevis""[Mesh:noexp] OR Saint Lucia[Mesh:noexp] OR ""Saint Vincent and the Grenadines""[Mesh:noexp] OR Samoa[Mesh:noexp] OR Saudi Arabia[Mesh:noexp] OR Senegal[Mesh:noexp] OR Serbia[Mesh:noexp] OR Montenegro[Mesh:noexp] OR Seychelles[Mesh:noexp] OR Sierra Leone[Mesh:noexp] OR Slovenia[Mesh:noexp] OR Sri Lanka[Mesh:noexp] OR Somalia[Mesh:noexp] OR South Africa[Mesh:noexp] OR Sudan[Mesh:noexp] OR Suriname[Mesh:noexp] OR Swaziland[Mesh:noexp] OR Syria[Mesh:noexp] OR Tajikistan[Mesh:noexp] OR Tanzania[Mesh:noexp] OR Thailand[Mesh:noexp] OR Togo[Mesh:noexp] OR Tonga[Mesh:noexp] OR ""Trinidad and Tobago""[Mesh:noexp] OR Tunisia[Mesh:noexp] OR Turkey[Mesh:noexp] OR Turkmenistan[Mesh:noexp] OR Uganda[Mesh:noexp] OR Ukraine[Mesh:noexp] OR Uruguay[Mesh:noexp] OR USSR[Mesh:noexp] OR Uzbekistan[Mesh:noexp] OR Vanuatu[Mesh:noexp] OR Venezuela[Mesh:noexp] OR Vietnam[Mesh:noexp] OR Yemen[Mesh:noexp] OR Yugoslavia[Mesh:noexp] OR Zambia[Mesh:noexp] OR Zimbabwe[Mesh:noexp]))

#7:
(mhealth[Title/Abstract] OR m-health[Title/Abstract] OR mobile health[Title/Abstract] OR telemedicine[Title/Abstract] OR telehealth[Title/Abstract] OR telecare[Title/Abstract] OR telehealth care[Title/Abstract] OR mobile telehealth care[Title/Abstract] OR mobile telemedicine[Title/Abstract] OR mcare[Title/Abstract] OR m-care[Title/Abstract] OR mobile communication[Title/Abstract] OR mobile technolog*[Title/Abstract] OR multimedia technolog*[Title/Abstract] OR mobile devic*[Title/Abstract] OR app[Title/Abstract] OR apps[Title/Abstract] OR mobile app*[Title/Abstract] OR portable electronic app*[Title/Abstract] OR portable software app*[Title/Abstract] OR voice messag*[Title/Abstract] OR website*[Title/Abstract] OR radio[Title/Abstract] OR cell phone*[Title/Abstract] OR cellular phone*[Title/Abstract] OR telephone*[Title/Abstract] OR cellular telephone*[Title/Abstract] OR portable cellular phone*[Title/Abstract] OR transportable cellular phone*[Title/Abstract] OR mobile phone*[Title/Abstract] OR smart phone*[Title/Abstract] OR smart-phone*[Title/Abstract] OR

smartphone*[Title/Abstract] OR mobile tablet computer*[Title/Abstract] OR tablet computer*[Title/Abstract] OR handheld computer*[Title/Abstract] OR microcomputer*[Title/Abstract] OR personal digital assistant*[Title/Abstract] OR PDA[Title/Abstract] OR text messag*[Title/Abstract] OR SMS[Title/Abstract] OR short messag*[Title/Abstract] OR multimedia messag*[Title/Abstract] OR multi-media messag*[Title/Abstract] OR podcast*[Title/Abstract] OR mp3[Title/Abstract] OR social media[Title/Abstract] OR social network*[Title/Abstract] OR facebook[Title/Abstract] OR twitter[Title/Abstract] OR twitter messag*[Title/Abstract] OR ehealth[Title/Abstract] OR e-health[Title/Abstract] OR telemedicine[MeSH Terms] OR short message service[MeSH Terms] OR cellular phone[MeSH Terms] OR mobile applications[MeSH Terms] OR text messaging[MeSH Terms] OR radio[MeSH Terms] OR computers, handheld[MeSH Terms] OR multimedia[MeSH Terms] OR social media[MeSH Terms] OR internet[MeSH Terms])

1.2 Search EMBASE (25 July 2014):

((#1 OR #2 OR (#3 AND #4) OR #5) AND #6 AND #7

#1:
pregnant:ab,ti OR expecting:ab,ti AND (women:ab,ti OR woman:ab,ti OR female:ab,ti) OR pregnancy:ab,ti OR mother*:ab,ti OR 'pregnant woman'/exp OR 'pregnant woman' OR 'midwife':ab,ti

#2:
'midwives':ab,ti OR 'lay midwife':ab,ti OR 'lay midwives':ab,ti OR 'traditional midwife':ab,ti OR 'traditional midwives':ab,ti OR 'nurse midwife':ab,ti OR 'nurse midwives':ab,ti OR 'birth attendant':ab,ti OR 'traditional birth attendant':ab,ti OR obstetrician*:ab,ti OR gynaecologist*:ab,ti OR gynecologist*:ab,ti OR 'nurse'/exp OR 'nurse' OR 'traditional birth attendant'/exp OR 'traditional birth attendant'

#3:
('health care provider':ab,ti OR 'health care providers':ab,ti OR 'community health worker':ab,ti OR 'community health workers':ab,ti OR nurse:ab,ti OR nurses:ab,ti OR 'nursing personnel':ab,ti OR 'nursing staff':ab,ti OR 'medical doctor':ab,ti OR 'medical doctors':ab,ti OR doctor*:ab,ti OR 'clinical officer':ab,ti OR 'clinical officers':ab,ti OR coh:ab,ti OR 'medical officer':ab,ti OR 'medical officers':ab,ti OR moh:ab,ti OR 'health personnel':ab,ti OR 'health staff':ab,ti OR 'health care personnel'/exp OR 'health care personnel' OR 'health auxillary' OR 'nurse'/exp OR 'nurse' OR 'nurses, public health'/exp OR 'nurses, public health'

#4:
maternal:ab,ti OR obstetric:ab,ti OR postpartum:ab,ti OR intrapartum:ab,ti OR newborn*:ab,ti OR antenatal:ab,ti OR prenatal:ab,ti OR postnatal:ab,ti OR perinatal:ab,ti OR infant*:ab,ti OR neonatal:ab,ti OR baby:ab,ti OR babies:ab,ti

#5:

maternal:ab,ti OR newborn*:ab,ti OR antenatal:ab,ti OR obstetric:ab,ti OR postnatal:ab,ti OR postpartum:ab,ti OR prenatal:ab,ti OR perinatal:ab,ti OR infant*:ab,ti OR intrapartum:ab,ti OR neonatal:ab,ti OR baby:ab,ti OR babies:ab,ti OR 'maternal care'/exp OR 'maternal care' OR 'obstetric procedure'/exp OR 'obstetric procedure' OR 'puerperium'/exp OR 'puerperium' OR 'newborn care'/exp OR 'newborn care' OR 'prenatal care'/exp OR 'prenatal care' OR 'postnatal care'/exp OR 'postnatal care'

#6:
('afghanistan'/exp OR 'albania'/exp OR 'algeria'/exp OR 'angola'/exp OR 'antigua'/exp OR barbuda OR 'argentina'/exp OR 'armenia'/exp OR armenian OR 'aruba'/exp OR 'azerbaijan'/exp OR 'bahrain'/exp OR 'bangladesh'/exp OR 'barbados'/exp OR 'benin'/exp OR 'byelarus'/exp OR byelorussian OR 'belarus'/exp OR belorussian OR belorussia OR 'belize'/exp OR 'bhutan'/exp OR 'bolivia'/exp OR bosnia OR herzegovina OR hercegovina OR 'botswana'/exp OR brasil OR 'brazil'/exp OR 'bulgaria'/exp OR 'burkina faso'/exp OR 'burkina fasso' OR 'upper volta'/exp OR 'burundi'/exp OR urundi OR 'cambodia'/exp OR 'khmer republic'/exp OR kampuchea OR 'cameroon'/exp OR cameroons OR cameron OR camerons OR 'cape verde'/exp OR 'central african republic'/exp OR 'chad'/exp OR 'chile'/exp OR 'china'/exp OR 'colombia'/exp OR 'comoros'/exp OR 'comoro islands'/exp OR comores OR 'mayotte'/exp OR 'congo'/exp OR 'zaire'/exp OR 'costa rica'/exp OR 'cote d ivoire'/exp OR 'ivory coast'/exp OR 'croatia'/exp OR 'cuba'/exp OR 'cyprus'/exp OR 'czechoslovakia'/exp OR 'czech republic'/exp OR 'slovakia'/exp OR 'slovak republic'/exp OR 'djibouti'/exp OR 'french somaliland' OR 'dominica'/exp OR 'dominican republic'/exp OR 'east timor'/exp OR 'east timur' OR 'timor leste'/exp OR 'ecuador'/exp OR 'egypt'/exp OR 'united arab republic'/exp OR 'el salvador'/exp OR 'eritrea'/exp OR 'estonia'/exp OR 'ethiopia'/exp OR 'fiji'/exp OR 'gabon'/exp OR 'gabonese republic' OR 'gambia'/exp OR gaza OR 'georgia republic' OR 'georgian republic' OR 'ghana'/exp OR 'gold coast' OR 'greece'/exp OR 'grenada'/exp OR 'guatemala'/exp OR 'guinea'/exp OR 'guam'/exp OR guiana OR 'guyana'/exp OR 'haiti'/exp OR 'honduras'/exp OR 'hungary'/exp OR 'india'/exp OR 'maldives'/exp OR 'indonesia'/exp OR 'iran'/exp OR 'iraq'/exp OR 'isle of man' OR 'jamaica'/exp OR 'jordan'/exp OR 'kazakhstan'/exp OR kazakh OR 'kenya'/exp OR 'kiribati'/exp OR 'korea'/exp OR 'kosovo'/exp OR 'kyrgyzstan'/exp OR kirghizia OR 'kyrgyz republic' OR kirghiz OR kirgizstan OR 'lao pdr' OR 'laos'/exp OR 'latvia'/exp OR 'lebanon'/exp OR 'lesotho'/exp OR 'basutoland'/exp OR 'liberia'/exp OR 'libya'/exp OR 'lithuania'/exp OR macedonia OR 'madagascar'/exp OR 'malagasy republic'/exp OR 'malaysia'/exp OR 'malaya'/exp OR malay OR sabah OR sarawak OR 'malawi'/exp OR nyasaland OR 'mali'/exp OR 'malta'/exp OR 'marshall islands'/exp OR 'mauritania'/exp OR 'mauritius'/exp OR 'agalega islands' OR 'mexico'/exp OR 'micronesia'/exp OR 'middle east'/exp OR 'moldova'/exp OR moldovia OR moldovian OR 'mongolia'/exp OR 'morocco'/exp OR ifni OR 'mozambique'/exp OR 'myanmar'/exp OR myanma OR 'burma'/exp OR 'namibia'/exp OR 'nepal'/exp OR 'netherlands antilles'/exp OR 'new caledonia'/exp OR 'nicaragua'/exp OR 'niger'/exp OR 'nigeria'/exp OR 'northern mariana islands'/exp OR 'oman'/exp OR muscat OR 'pakistan'/exp OR 'palau'/exp OR 'palestine'/exp OR 'panama'/exp OR 'paraguay'/exp OR 'peru'/exp OR

'philippines'/exp OR philipines OR phillipines OR phillippines OR 'poland'/exp OR 'portugal'/exp OR 'puerto rico'/exp OR 'romania'/exp OR 'rumania'/exp OR roumania OR 'russia'/exp OR russian OR 'rwanda'/exp OR ruanda OR 'saint kitts' OR 'st kitts' OR 'nevis'/exp OR 'saint lucia'/exp OR 'st lucia'/exp OR 'saint vincent' OR 'st vincent' OR grenadines OR 'samoa'/exp OR 'samoan islands'/exp OR 'navigator island' OR 'navigator islands' OR 'sao tome' OR 'saudi arabia'/exp OR 'senegal'/exp OR 'serbia'/exp OR 'montenegro'/exp OR 'seychelles'/exp OR 'sierra leone'/exp OR 'slovenia'/exp OR 'sri lanka'/exp OR 'ceylon'/exp OR 'solomon islands'/exp OR 'somalia'/exp OR 'south africa'/exp OR 'sudan'/exp OR 'suriname'/exp OR 'surinam'/exp OR 'swaziland'/exp OR 'syria'/exp OR 'tajikistan'/exp OR tadzhikistan OR tadjikistan OR tadzhik OR 'tanzania'/exp OR 'thailand'/exp OR 'togo'/exp OR 'togolese republic' OR 'tonga'/exp OR trinidad OR tobago OR 'tunisia'/exp OR turkey OR 'turkmenistan'/exp OR turkmen OR 'uganda'/exp OR 'ukraine'/exp OR 'uruguay'/exp OR 'ussr'/exp OR 'soviet union'/exp OR 'union of soviet socialist republics' OR 'uzbekistan'/exp OR uzbek OR 'vanuatu'/exp OR 'new hebrides' OR 'venezuela'/exp OR 'vietnam'/exp OR 'viet nam'/exp OR 'west bank' OR 'yemen'/exp OR 'yugoslavia'/exp OR 'zambia'/exp OR 'zimbabwe'/exp OR 'rhodesia'/exp OR afghanistan:ab,ti OR albania:ab,ti OR algeria:ab,ti OR angola:ab,ti OR antigua:ab,ti OR barbuda:ab,ti OR argentina:ab,ti OR armenia:ab,ti OR armenian:ab,ti OR aruba:ab,ti OR azerbaijan:ab,ti OR bahrain:ab,ti OR bangladesh:ab,ti OR barbados:ab,ti OR benin:ab,ti OR byelarus:ab,ti OR byelorussian:ab,ti OR belarus:ab,ti OR belorussian:ab,ti OR belorussia:ab,ti OR belize:ab,ti OR bhutan:ab,ti OR bolivia:ab,ti OR bosnia:ab,ti OR herzegovina:ab,ti OR hercegovina:ab,ti OR botswana:ab,ti OR brasil:ab,ti OR brazil:ab,ti OR bulgaria:ab,ti OR 'burkina faso':ab,ti OR 'burkina fasso':ab,ti OR 'upper volta':ab,ti OR burundi:ab,ti OR urundi:ab,ti OR cambodia:ab,ti OR 'khmer republic':ab,ti OR kampuchea:ab,ti OR cameroon:ab,ti OR cameroons:ab,ti OR cameron:ab,ti OR camerons:ab,ti OR 'cape verde':ab,ti OR 'central african republic':ab,ti OR chad:ab,ti OR chile:ab,ti OR china:ab,ti OR colombia:ab,ti OR comoros:ab,ti OR 'comoro islands':ab,ti OR comores:ab,ti OR mayotte:ab,ti OR congo:ab,ti OR zaire:ab,ti OR 'costa rica':ab,ti OR 'cote d ivoire':ab,ti OR 'ivory coast':ab,ti OR croatia:ab,ti OR cuba:ab,ti OR cyprus:ab,ti OR czechoslovakia:ab,ti OR 'czech republic':ab,ti OR slovakia:ab,ti OR 'slovak republic':ab,ti OR djibouti:ab,ti OR 'french somaliland':ab,ti OR dominica:ab,ti OR 'dominican republic':ab,ti OR 'east timor':ab,ti OR 'east timur':ab,ti OR 'timor leste':ab,ti OR ecuador:ab,ti OR egypt:ab,ti OR 'united arab republic':ab,ti OR 'el salvador':ab,ti OR eritrea:ab,ti OR estonia:ab,ti OR ethiopia:ab,ti OR fiji:ab,ti OR gabon:ab,ti OR 'gabonese republic':ab,ti OR gambia:ab,ti OR gaza:ab,ti OR 'georgia republic':ab,ti OR 'georgian republic':ab,ti OR ghana:ab,ti OR 'gold coast':ab,ti OR greece:ab,ti OR grenada:ab,ti OR guatemala:ab,ti OR guinea:ab,ti OR guam:ab,ti OR guiana:ab,ti OR guyana:ab,ti OR haiti:ab,ti OR honduras:ab,ti OR hungary:ab,ti OR india:ab,ti OR maldives:ab,ti OR indonesia:ab,ti OR iran:ab,ti OR iraq:ab,ti OR 'isle of man':ab,ti OR jamaica:ab,ti OR jordan:ab,ti OR kazakhstan:ab,ti OR kazakh:ab,ti OR kenya:ab,ti OR kiribati:ab,ti OR korea:ab,ti OR kosovo:ab,ti OR kyrgyzstan:ab,ti OR kirghizia:ab,ti OR 'kyrgyz republic':ab,ti OR kirghiz:ab,ti OR kirgizstan:ab,ti OR 'lao pdr':ab,ti OR laos:ab,ti OR latvia:ab,ti OR lebanon:ab,ti OR lesotho:ab,ti OR basutoland:ab,ti OR liberia:ab,ti OR libya:ab,ti OR lithuania:ab,ti

OR macedonia:ab,ti OR madagascar:ab,ti OR 'malagasy republic':ab,ti OR malaysia:ab,ti OR malaya:ab,ti OR malay:ab,ti OR sabah:ab,ti OR sarawak:ab,ti OR malawi:ab,ti OR nyasaland:ab,ti OR mali:ab,ti OR malta:ab,ti OR 'marshall islands':ab,ti OR mauritania:ab,ti OR mauritius:ab,ti OR 'agalega islands':ab,ti OR mexico:ab,ti OR micronesia:ab,ti OR 'middle east':ab,ti OR moldova:ab,ti OR moldovia:ab,ti OR moldovian:ab,ti OR mongolia:ab,ti OR morocco:ab,ti OR ifni:ab,ti OR mozambique:ab,ti OR myanmar:ab,ti OR myanma:ab,ti OR burma:ab,ti OR namibia:ab,ti OR nepal:ab,ti OR 'netherlands antilles':ab,ti OR 'new caledonia':ab,ti OR nicaragua:ab,ti OR niger:ab,ti OR nigeria:ab,ti OR 'northern mariana islands':ab,ti OR oman:ab,ti OR muscat:ab,ti OR pakistan:ab,ti OR palau:ab,ti OR palestine:ab,ti OR panama:ab,ti OR paraguay:ab,ti OR peru:ab,ti OR philippines:ab,ti OR philipines:ab,ti OR phillipines:ab,ti OR phillippines:ab,ti OR poland:ab,ti OR portugal:ab,ti OR 'puerto rico':ab,ti OR romania:ab,ti OR rumania:ab,ti OR roumania:ab,ti OR russia:ab,ti OR russian:ab,ti OR rwanda:ab,ti OR ruanda:ab,ti OR 'saint kitts':ab,ti OR 'st kitts':ab,ti OR nevis:ab,ti OR 'saint lucia':ab,ti OR 'st lucia':ab,ti OR 'saint vincent':ab,ti OR 'st vincent':ab,ti OR grenadines:ab,ti OR samoa:ab,ti OR 'samoan islands':ab,ti OR 'navigator island':ab,ti OR 'navigator islands':ab,ti OR 'sao tome':ab,ti OR 'saudi arabia':ab,ti OR senegal:ab,ti OR serbia:ab,ti OR montenegro:ab,ti OR seychelles:ab,ti OR 'sierra leone':ab,ti OR slovenia:ab,ti OR 'sri lanka':ab,ti OR ceylon:ab,ti OR 'solomon islands':ab,ti OR somalia:ab,ti OR 'south africa':ab,ti OR sudan:ab,ti OR suriname:ab,ti OR surinam:ab,ti OR swaziland:ab,ti OR syria:ab,ti OR tajikistan:ab,ti OR tadzhikistan:ab,ti OR tadjikistan:ab,ti OR tadzhik:ab,ti OR tanzania:ab,ti OR thailand:ab,ti OR togo:ab,ti OR 'togolese republic':ab,ti OR tonga:ab,ti OR trinidad:ab,ti OR tobago:ab,ti OR tunisia:ab,ti OR turkey:ab,ti OR turkmenistan:ab,ti OR turkmen:ab,ti OR uganda:ab,ti OR ukraine:ab,ti OR uruguay:ab,ti OR ussr:ab,ti OR 'soviet union':ab,ti OR 'union of soviet socialist republics':ab,ti OR uzbekistan:ab,ti OR uzbek:ab,ti OR vanuatu:ab,ti OR 'new hebrides':ab,ti OR venezuela:ab,ti OR vietnam:ab,ti OR 'viet nam':ab,ti OR 'west bank':ab,ti OR yemen:ab,ti OR yugoslavia:ab,ti OR zambia:ab,ti OR zimbabwe:ab,ti OR rhodesia:ab,ti OR ('emerging country':ab,ti OR 'emerging countries':ab,ti OR low:ab,ti OR limited:ab,ti OR constrained:ab,ti OR restricted:ab,ti OR delimited:ab,ti OR restrained:ab,ti AND resource:ab,ti) OR 'developing country':ab,ti OR 'developing countries':ab,ti OR 'developing nation':ab,ti OR 'developing nations':ab,ti OR 'developing population':ab,ti OR 'developing populations':ab,ti OR 'developing world':ab,ti OR 'less developed country':ab,ti OR 'less developed countries':ab,ti OR 'less developed nation':ab,ti OR 'less developed nations':ab,ti OR 'less developed population':ab,ti OR 'less developed populations':ab,ti OR 'less developed world':ab,ti OR 'lesser developed country':ab,ti OR 'lesser developed countries':ab,ti OR 'lesser developed nation':ab,ti OR 'lesser developed nations':ab,ti OR 'lesser developed population':ab,ti OR 'lesser developed populations':ab,ti OR 'lesser developed world':ab,ti OR 'under developed country':ab,ti OR 'under developed countries':ab,ti OR 'under developed nation':ab,ti OR 'under developed nations':ab,ti OR 'under developed population':ab,ti OR 'under developed populations':ab,ti OR 'under developed world':ab,ti OR 'underdeveloped country':ab,ti OR 'underdeveloped countries':ab,ti OR 'underdeveloped

nation':ab,ti OR 'underdeveloped nations':ab,ti OR 'underdeveloped population':ab,ti OR 'underdeveloped populations':ab,ti OR 'underdeveloped world':ab,ti OR 'middle income country':ab,ti OR 'middle income countries':ab,ti OR 'middle income nation':ab,ti OR 'middle income nations':ab,ti OR 'middle income population':ab,ti OR 'middle income populations':ab,ti OR 'low income country':ab,ti OR 'low income countries':ab,ti OR 'low income nation':ab,ti OR 'low income nations':ab,ti OR 'low income population':ab,ti OR 'low income populations':ab,ti OR 'lower income country':ab,ti OR 'lower income countries':ab,ti OR 'lower income nation':ab,ti OR 'lower income nations':ab,ti OR 'lower income population':ab,ti OR 'lower income populations':ab,ti OR 'underserved country':ab,ti OR 'underserved countries':ab,ti OR 'underserved nation':ab,ti OR 'underserved nations':ab,ti OR 'underserved population':ab,ti OR 'underserved populations':ab,ti OR 'underserved world':ab,ti OR 'under served country':ab,ti OR 'under served countries':ab,ti OR 'under served nation':ab,ti OR 'under served nations':ab,ti OR 'under served population':ab,ti OR 'under served populations':ab,ti OR 'under served world':ab,ti OR 'deprived country':ab,ti OR 'deprived countries':ab,ti OR 'deprived nation':ab,ti OR 'deprived nations':ab,ti OR 'deprived population':ab,ti OR 'deprived populations':ab,ti OR 'deprived world':ab,ti OR 'poor country':ab,ti OR 'poor countries':ab,ti OR 'poor nation':ab,ti OR 'poor nations':ab,ti OR 'poor population':ab,ti OR 'poor populations':ab,ti OR 'poor world':ab,ti OR 'poorer country':ab,ti OR 'poorer countries':ab,ti OR 'poorer nation':ab,ti OR 'poorer nations':ab,ti OR 'poorer population':ab,ti OR 'poorer populations':ab,ti OR 'poorer world':ab,ti OR 'developing economy':ab,ti OR 'developing economies':ab,ti OR 'less developed economy':ab,ti OR 'less developed economies':ab,ti OR 'lesser developed economy':ab,ti OR 'lesser developed economies':ab,ti OR 'under developed economy':ab,ti OR 'under developed economies':ab,ti OR 'underdeveloped economy':ab,ti OR 'underdeveloped economies':ab,ti OR 'middle income economy':ab,ti OR 'middle income economies':ab,ti OR 'low income economy':ab,ti OR 'low income economies':ab,ti OR 'lower income economy':ab,ti OR 'lower income economies':ab,ti OR 'low gdp':ab,ti OR 'low gnp':ab,ti OR 'low gross domestic':ab,ti OR 'low gross national':ab,ti OR 'lower gdp':ab,ti OR 'lower gnp':ab,ti OR 'lower gross domestic':ab,ti OR 'lower gross national':ab,ti OR lmic:ab,ti OR lmics:ab,ti OR 'third world':ab,ti OR 'lami country':ab,ti OR 'lami countries':ab,ti OR 'transitional country':ab,ti OR 'transitional countries':ab,ti OR 'poverty'/exp OR 'poverty' OR africa:ab,ti OR asia:ab,ti OR caribbean:ab,ti OR 'west indies':ab,ti OR 'south america':ab,ti OR 'latin america':ab,ti OR 'central america':ab,ti OR 'developing country'/exp OR 'developing country')

#7:
(mhealth:ab,ti OR 'm health':ab,ti OR 'mobile health':ab,ti OR ehealth:ab,ti OR 'e health':ab,ti OR telemedicine:ab,ti OR telehealth:ab,ti OR telecare:ab,ti OR 'telehealth care':ab,ti OR 'mobile telehealth care':ab,ti OR 'mobile telemedicine':ab,ti OR mcare:ab,ti OR 'm care':ab,ti OR 'mobile communication':ab,ti OR 'mobile technology':ab,ti OR 'mobile technologies':ab,ti OR 'multimedia technology':ab,ti OR 'multimedia technologies':ab,ti OR 'mobile device':ab,ti OR 'mobile devices':ab,ti OR 'app':ab,ti OR 'apps':ab,ti OR 'mobile

application':ab,ti OR 'mobile applications':ab,ti OR 'mobile app':ab,ti OR 'mobile apps':ab,ti OR 'portable electronic app':ab,ti OR 'portable electronic apps':ab,ti OR 'portable electronic application':ab,ti OR 'portable electronic applications':ab,ti OR 'portable software app':ab,ti OR 'portable software apps':ab,ti OR 'portable software application':ab,ti OR 'portable software applications':ab,ti OR 'voice message':ab,ti OR 'voice messages':ab,ti OR radio:ab,ti OR 'cell phone':ab,ti OR 'cell phones':ab,ti OR 'cellular phone':ab,ti OR 'cellular phones':ab,ti OR telephone*:ab,ti OR 'cellular telephone':ab,ti OR 'cellular telephones':ab,ti OR 'portable cellular phone':ab,ti OR 'portable cellular phones':ab,ti OR 'transportable cellular phone':ab,ti OR 'transportable cellular phones':ab,ti OR 'mobile phone':ab,ti OR 'mobile phones':ab,ti OR 'smart phone':ab,ti OR 'smart phones':ab,ti OR smartphone*:ab,ti OR 'smart-phone':ab,ti OR 'smart-phones':ab,ti OR 'mobile tablet computer':ab,ti OR 'mobile tablet computers':ab,ti OR 'tablet computer':ab,ti OR 'tablet computers':ab,ti OR 'handheld computer':ab,ti OR 'handheld computers':ab,ti OR microcomputer*:ab,ti OR 'personal digital assistant':ab,ti OR 'personal digital assistants':ab,ti OR pda:ab,ti OR 'text message':ab,ti OR 'text messages':ab,ti OR sms:ab,ti OR 'short message service':ab,ti OR 'short message services':ab,ti OR 'multimedia message':ab,ti OR 'multimedia messages':ab,ti OR 'multi-media message':ab,ti OR 'multi-media messages':ab,ti OR podcast*:ab,ti OR mp3:ab,ti OR 'social media':ab,ti OR 'social network':ab,ti OR 'social networks':ab,ti OR facebook:ab,ti OR twitter:ab,ti OR 'twitter message':ab,ti OR 'twitter messages':ab,ti OR 'telemedicine'/exp OR 'telemedicine') NOT 'patent ductus arteriosus':ab,ti

1.3 Search Cochrane (25 July 2014):

((#1 OR #2 OR (#3 AND #4) OR #5) AND #6 AND #7

#1:
(pregnant:ti,ab or expecting:ti,ab AND women:ti,ab or woman:ti,ab or female:ti,ab) OR (pregnancy:ti,ab or mother*:ti,ab) OR MeSH descriptor: [Pregnant Women] this term only

#2:
(midwife:ti,ab or midwives:ti,ab or "lay midwife":ti,ab or "lay midwives":ti,ab or "traditional midwife":ti,ab or "traditional midwives":ti,ab or "nurse midwife":ti,ab or "nurse midwives":ti,ab or "birth attendant*":ti,ab or "traditional birth attendant*":ti,ab or obstetrician*:ti,ab or gynaecologist*:ti,ab or gynecologist*:ti,ab) OR MeSH descriptor: [Nurse Midwives] explode all trees OR MeSH descriptor: [Midwifery] explode all trees

#3:
(health care provider*:ti,ab or "community health worker*":ti,ab or nurse*:ti,ab or "nursing personnel":ti,ab or "nursing staff":ti,ab or "medical doctor*":ti,ab or doctor*:ti,ab or "clinical officer*":ti,ab or COH:ti,ab or "medical officer*":ti,ab or MOH:ti,ab or "health personnel":ti,ab or "health staff":ti,ab) OR MeSH descriptor: [Health Personnel] explode all trees OR MeSH descriptor: [Community Health

Workers] explode all trees OR MeSH descriptor: [Nurses, Public Health] explode all trees OR MeSH descriptor: [Nurses] explode all trees

#4:
maternal:ti,ab or newborn*:ti,ab or antenatal:ti,ab or obstetric:ti,ab or postnatal:ti,ab or postpartum:ti,ab or prenatal:ti,ab or perinatal:ti,ab or infant*:ti,ab or intrapartum:ti,ab or neonatal:ti,ab or baby:ti,ab or babies:ti,ab

#5:
(maternal:ti,ab or newborn*:ti,ab or antenatal:ti,ab or obstetric:ti,ab or postnatal:ti,ab or postpartum:ti,ab or prenatal:ti,ab or perinatal:ti,ab or infant*:ti,ab or intrapartum:ti,ab or neonatal:ti,ab or baby:ti,ab or babies:ti,ab) OR MeSH descriptor: [Maternal-Child Nursing] explode all trees OR MeSH descriptor: [Maternal Health Services] explode all trees OR MeSH descriptor: [Delivery, Obstetric] explode all trees OR MeSH descriptor: [Obstetrics] explode all trees OR MeSH descriptor: [Postnatal Care] explode all trees OR MeSH descriptor: [Prenatal Care] explode all trees OR MeSH descriptor: [Perinatal Care] this term only OR MeSH descriptor: [Neonatal Nursing] explode all trees

#6:
(Africa or Asia or Caribbean or "West Indies" or "South America" or "Latin America" or "Central America"):ti,ab,kw OR (Afghanistan or Albania or Algeria or Angola or Antigua or Barbuda or Argentina or Armenia or Armenian or Aruba or Azerbaijan or Bahrain or Bangladesh or Barbados or Benin or Byelarus or Byelorussian or Belarus or Belorussian or Belorussia or Belize or Bhutan or Bolivia or Bosnia or Herzegovina or Hercegovina or Botswana or Brasil or Brazil or Bulgaria or "Burkina Faso" or "Burkina Fasso" or "Upper Volta" or Burundi or Urundi or Cambodia or "Khmer Republic" or Kampuchea or Cameroon or Cameroons or Cameron or Camerons or "Cape Verde" or "Central African Republic" or Chad or Chile or China or Colombia or Comoros or "Comoro Islands" or Comores or Mayotte or Congo or Zaire or "Costa Rica" or "Cote d'Ivoire" or "Ivory Coast" or Croatia or Cuba or Cyprus or Czechoslovakia or "Czech Republic" or Slovakia or "Slovak Republic"):ti,ab,kw OR (Djibouti or "French Somaliland" or Dominica or "Dominican Republic" or "East Timor" or "East Timur" or "Timor Leste" or Ecuador or Egypt or "United Arab Republic" or "El Salvador" or Eritrea or Estonia or Ethiopia or Fiji or Gabon or "Gabonese Republic" or Gambia or Gaza or Georgia or Georgian or Ghana or "Gold Coast" or Greece or Grenada or Guatemala or Guinea or Guam or Guiana or Guyana or Haiti or Honduras or Hungary or India or Maldives or Indonesia or Iran or Iraq or "Isle of Man" or Jamaica or Jordan or Kazakhstan or Kazakh or Kenya or Kiribati or Korea or Kosovo or Kyrgyzstan or Kirghizia or "Kyrgyz Republic" or Kirghiz or Kirgizstan or "Lao PDR" or Laos or Latvia or Lebanon or Lesotho or Basutoland or Liberia or Libya or Lithuania):ti,ab,kw OR (Macedonia or Madagascar or "Malagasy Republic" or Malaysia or Malaya or Malay or Sabah or Sarawak or Malawi or Nyasaland or Mali or Malta or "Marshall Islands" or Mauritania or Mauritius or "Agalega Islands" or Mexico or Micronesia or "Middle East" or Moldova or Moldovia or Moldovian or Mongolia or Montenegro or Morocco or Ifni or Mozambique or Myanmar or

Myanma or Burma or Namibia or Nepal or "Netherlands Antilles" or "New Caledonia" or Nicaragua or Niger or Nigeria or "Northern Mariana Islands" or Oman or Muscat or Pakistan or Palau or Palestine or Panama or Paraguay or Peru or Philippines or Philipines or Phillipines or Phillippines or Poland or Portugal or "Puerto Rico"):ti,ab,kw OR (Romania or Rumania or Roumania or Russia or Russian or Rwanda or Ruanda or "Saint Kitts" or "St Kitts" or Nevis or "Saint Lucia" or "St Lucia" or "Saint Vincent" or "St Vincent" or Grenadines or Samoa or "Samoan Islands" or "Navigator Island" or "Navigator Islands" or "Sao Tome" or "Saudi Arabia" or Senegal or Serbia or Montenegro or Seychelles or "Sierra Leone" or Slovenia or "Sri Lanka" or Ceylon or "Solomon Islands" or Somalia or Sudan or Suriname or Surinam or Swaziland or Syria or Tajikistan or Tadzhikistan or Tadjikistan or Tadzhik or Tanzania or Thailand or Togo or "Togolese Republic" or Tonga or Trinidad or Tobago or Tunisia or Turkey or Turkmenistan or Turkmen or Uganda or Ukraine or Uruguay or USSR or "Soviet Union" or "Union of Soviet Socialist Republics" or Uzbekistan or Uzbek or Vanuatu or "New Hebrides" or Venezuela or Vietnam or "Viet Nam" or "West Bank" or Yemen or Yugoslavia or Zambia or Zimbabwe or Rhodesia):ti,ab,kw OR (developing or less* next developed or "under developed" or underdeveloped or "middle income" or low* next income or underserved or "under served" or deprived or poor*) next (countr* or nation* or population* or world):ti,ab,kw OR (developing or less* next developed or "under developed" or underdeveloped or "middle income" or low* next income) next (economy or economies):ti,ab,kw OR low* next (gdp or gnp or "gross domestic" or "gross national"):ti,ab,kw OR (low near/3 middle near/3 countr*):ti,ab,kw OR (lmic or lmics or "third world" or "lami country" or "lami countries"):ti,ab,kw OR ("transitional country" or "transitional countries"):ti,ab,kw OR ("low resource" or " limited resource" or "constrained resource" or "restricted resource" or "delimited resource" or "restrained resource" or "emerging country" or "emerging countries"):ti,ab,kw

#7:
(mhealth:ti,ab or m-health:ti,ab or "mobile health":ti,ab or "telemedicine":ti,ab or telehealth:ti,ab or telecare:ti,ab or "telehealth care":ti,ab or "mobile telehealth care":ti,ab or "mobile telemedicine":ti,ab or mcare:ti,ab or m-care:ti,ab or "mobile communication":ti,ab or "mobile technolog*":ti,ab or "multimedia technolog*":ti,ab or "mobile devic*":ti,ab or app:ti,ab or apps:ti,ab or "mobile app*":ti,ab or "portable electronic app*":ti,ab or "portable software app*":ti,ab or "voice messag*":ti,ab or website*:ti,ab or radio:ti,ab or "cell phone*":ti,ab or "cellular phone*":ti,ab or telephone*:ti,ab or "cellular telephone*":ti,ab or "portable cellular phone*":ti,ab or "transportable cellular phone*":ti,ab or "mobile phone*":ti,ab or "smart phone*":ti,ab or smart-phone*:ti,ab or smartphone*:ti,ab or "mobile tablet computer*":ti,ab or "tablet computer*":ti,ab or "handheld computer*":ti,ab or microcomputer*:ti,ab or "personal digital assistant*":ti,ab or PDA:ti,ab or "text messag*":ti,ab or SMS:ti,ab or "short messag*":ti,ab or "multimedia messag*":ti,ab or "multi-media messag*":ti,ab or podcast*:ti,ab or mp3:ti,ab or "social media":ti,ab or "social network*":ti,ab or facebook:ti,ab or twitter:ti,ab or "twitter messag*":ti,ab or ehealth:ti,ab or e-health:ti,ab) OR MeSH descriptor: [Telemedicine] explode all trees OR MeSH descriptor: [Cellular Phone]

this term only OR MeSH descriptor: [Text Messaging] this term only OR MeSH descriptor: [Microcomputers] explode all trees OR MeSH descriptor: [Mobile Applications] this term only

1.4 Search: Global Health Library (30 July 2014):

("pregnant women" OR "midwife" OR "midwives" OR "birth attendant" OR "birth attendants" OR "obstetrician" OR "obstetricians" OR "gynaecologist" OR "gynaecologists" OR "gynecologist" OR "gynecologists" OR "community health worker" OR "community health workers" OR "nurse" OR "nurses" OR "doctor" OR "doctors" OR "clinical officer" OR "clinical officers" OR "medical officer" OR "medical officers" OR "maternal" OR "newborn"") AND (developing country" OR "developing countries" OR "low income country" OR "low income countries" OR "middle income country" OR "middle income countries" OR "low resource") AND ("mhealth" OR "mobile health" OR "m-health")

1.5 Search Popline (30 July 2014):

("pregnant women" OR "midwife" OR "midwives" OR "birth attendant" OR "birth attendants" OR "obstetrician" OR "obstetricians" OR "gynaecologist" OR "gynaecologists" OR "gynecologist" OR "gynecologists" OR "community health worker" OR "community health workers" OR "nurse" OR "nurses" OR "doctor" OR "doctors" OR "clinical officer" OR "clinical officers" OR "medical officer" OR "medical officers" OR "maternal" OR "newborn"") AND (developing country" OR "developing countries" OR "low income country" OR "low income countries" OR "middle income country" OR "middle income countries" OR "low resource") AND ("mhealth" OR "mobile health" OR "m-health")
