# Supplementary material for: Effectiveness of mHealth Interventions Targeting Health Care Workers to Improve Pregnancy Outcomes in Low- and Middle-Income Countries: A Systematic Review
Source: J Med Internet Res. 2016 Aug 19;18(8):e226. doi: 10.2196/jmir.5533 (PMC5010646; doi:10.2196/jmir.5533)
Supplement: Multimedia Appendix 3 [file jmir_v18i8e226_app3.pdf]

Supplement 3: Adapted quality assessment tool

Risk category	Low risk	High risk	Unclear risk
**Blinding: (detection bias)**	•No blinding or incomplete blinding, but the review authors judge that the outcome is not likely to be influenced by lack of blinding;	•No blinding or incomplete blinding, and the outcome is likely to be influenced by lack of blinding;	•Insufficient information to permit judgment of 'Low risk' or 'High risk';
	•Blinding of participants and key study personnel ensured, and unlikely that the blinding could have been broken.	•Blinding of key study participants and personnel attempted, but likely that the blinding could have been broken, and the outcome is likely to be influenced by lack of blinding.	•The study did not address this outcome.
**Selection of study population: (selection bias)**	•The individuals selected to participate are representative of the target population	•The individuals selected to participate are somewhat likely/not likely to be representative of the target population	•Not described whether individuals selected to participate in the study are likely to be representative of the target population
	•The investigators describe a random component in the sequence generation process	•The investigators describe a non-random component in the sequence generation process.	•Insufficient information about the sequence generation process to permit judgement of 'Yes' or 'No'.
Case-control specific:	•Case definition is adequate with independent validation	•Case definition is adequate, e.g. record linkage or based on self-reports	•No description of the case definition
	•Consecutive or obviously representative series of cases	•Potential for selection biases	•Representativeness of cases not stated
	•Selection of controls occurred from community controls with no history of disease	•Controls are hospital controls	•There is no description of the controls
Cohort specific:	•Representativeness of the exposed cohort is truly representative of the average …(describe) in the community	•Representativeness of the exposed cohort is somewhat representative of the average …(describe) in the community	•There is no description of the derivation of the cohort
	•The non-exposed cohort is drawn from the same community as the exposed cohort	•The non-exposed cohort is drawn from a different source	•There is no description of the derivation of the non-exposed cohort

**Completeness: (attrition bias)**	•No missing outcome data;	•Reason for missing outcome data likely to be related to true outcome, with either imbalance in numbers or reasons for missing data across intervention groups;	•Insufficient reporting of attrition/exclusions to permit judgement of 'Low risk' or 'High risk' (e.g. number randomized not stated, no reasons for missing data provided);
	•Reasons for missing outcome data unlikely to be related to true outcome (for survival data, censoring unlikely to be introducing bias);	•For dichotomous outcome data, the proportion of missing outcomes compared with observed event risk enough to induce clinically relevant bias in intervention effect estimate;	•The study did not address this outcome
	•Missing outcome data balanced in numbers across intervention groups, with similar reasons for missing data across groups;	•For continuous outcome data, plausible effect size (difference in means or standardized difference in means) among missing outcomes enough to induce clinically relevant bias in observed effect size;	
	•For dichotomous outcome data, the proportion of missing outcomes compared with observed event risk not enough to have a clinically relevant impact on the intervention effect estimate;	•'As-treated' analysis done with substantial departure of the intervention received from that assigned at randomization;	
	•For continuous outcome data, plausible effect size (difference in means or standardized difference in means) among missing outcomes not enough to have a clinically relevant impact on observed effect size;	•Potentially inappropriate application of simple imputation	
	•Missing data have been imputed using appropriate methods	•Lost to follow up is mentioned but there is a large size variation between the patient and control group.	
	•Lost to follow up is mentioned and is comparable within the patient as well as the control group.		
Case-control specific:	•Non-response rate was same rate for both groups	•Non respondents described	•Non-response rate different and no designation
**Origin:**	•Self-measurements or data was gathered by adequate personnel (midwife, research assistant etc.)	•Data from database (not collected by researches themselves)	•Article does not describe where data came from.
Cohort specific:	•Assessment of outcome occurred through independent blind assessment and/or record linkage	•Assessment of outcome occurred through self-report	•No description of assessment of outcome
**Definition of outcome:**	•Article gives adequate definitions of outcome measurements	•Article does not give adequate definitions of outcome measurements	•Article does not give adequate definitions for ALL outcome measurements

<table>
<tr><td></td><td></td><td></td><td></td></tr>
<tr><td rowspan="2">Confounders:</td><td rowspan="2">•Article states that confounders were taken into account and define the confounders</td><td rowspan="2">•Article states that confounders were not taken into account.</td><td>•Article only states that confounders were taken into account, but no specific confounders are given</td></tr>
<tr><td>•Article does not state that confounders are taken into account</td></tr>
</table>
