# Supplementary material for: Effectiveness of mHealth Interventions Targeting Health Care Workers to Improve Pregnancy Outcomes in Low- and Middle-Income Countries: A Systematic Review
Source: J Med Internet Res. 2016 Aug 19;18(8):e226. doi: 10.2196/jmir.5533 (PMC5010646; doi:10.2196/jmir.5533)
Supplement: Multimedia Appendix 4 [file jmir_v18i8e226_app4.pdf]

## Supplement 4: Table Showing Risk of bias assessment for included intervention studies

| Study | Blinding of researcher /clinician | Selection of study population | Completeness of data | Origin of data (database of measurements) | Clear definition of outcome? | Confounders taken into account? |
|---|---|---|---|---|---|---|
| 2014 Munro et al. | NA | Non-randomized Participants selected based on a criteria but no random process **High risk** | No missing data reported **Low risk** | Primary data collection Data assessed for reliability and validity **Low risk** | Yes **Low risk** | Not clearly stated **Unclear risk** |
| 2014 Pathfinder (Grey Literature) | NA | Non randomized trial **High risk** | No missing data reported **Low risk** | Primary data collection by trained interviewers. Monitoring done **Low risk** | Yes **Low risk** | Not clearly stated **Unclear risk** |
| 2013 Little et al | NA | Non-randomized **High risk** | No missing data reported **Low risk** | Primary data collection **Low risk** | Yes **Low risk** | Not clearly stated **Unclear risk** |
| 2012 Zhang et al. | NA | Randomization of respondents Control group used **Low risk** | Errors checked for No missing data reported **Low risk** | Primary data collection **Low risk** | Yes **Low risk** | Not clearly stated **Unclear risk** |
| 2012 Lori et al. | NA | Non-randomized **High risk** | No missing data reported **Low Risk** | Primary data collection **Low risk** | Yes **Low risk** | Age, sex, and whether anyone in her family owned a cell phone were account for **Low risk** |
| 2012 Seidenberg et al. | NA | No description of random component **High risk** | No missing data reported **Low risk** | Primary data collection **Low risk** | Yes **Low risk** | Confounding factors not taken into account. Outcomes possibly affected by observer expectancy |

| | | | | | | |
|---|---|---|---|---|---|---|
| | | | | | | Benefits of mhealth possibly underestimated **High risk** |
| 2012 Lemay et al. | NA | Non randomized Random sampling Use of a control group **Low risk** | No missing data reported **Low risk** | Primary data collection **Low risk** | Yes **Low risk** | Not clearly stated **Unclear risk** |
| 2012 Ngabo et al | NA | Not clearly stated **Unclear risk** | No missing data reported **Low risk** | Primary data collection **Low risk** | Yes **Low risk** | Not clearly stated **Unclear risk** |
| 2011 Andreatta et al. | NA | Non-randomized Small purposively selected sample **High risk** | Uncertainty of data quality, no secondary monitoring **High risk** | Primary data collection **Low risk** | Yes **Low risk** | Not clearly stated **Unclear risk** |
| 2010 Kaewkungwal et al. | NA | Non-randomized No control group **High risk** | No missing data reported **High risk** | Primary data collection **Low risk** | Yes **Low risk** | Confounding factors not taken into account **High risk** |

NA: not applicable due to study design
